# Evaluating short-term forecasting of COVID-19 cases among different epidemiological models under a Bayesian framework

**DOI:** 10.1093/gigascience/giab009

**Published:** 2021-02-19

**Authors:** Qiwei Li, Tejasv Bedi, Christoph U Lehmann, Guanghua Xiao, Yang Xie

**Affiliations:** Department of Mathematical Sciences, The University of Texas at Dallas, 800 W Campbell Rd, Richardson, TX 75080, USA; Department of Mathematical Sciences, The University of Texas at Dallas, 800 W Campbell Rd, Richardson, TX 75080, USA; Department of Pediatrics, The University of Texas Southwestern Medical Center, Dallas, TX 75390, USA; Lyda Hill Department of Bioinformatics, The University of Texas Southwestern Medical Center, Dallas, TX 75390, USA; Department of Population and Data Sciences, The University of Texas Southwestern Medical Center, Dallas, TX 75390, USA; Lyda Hill Department of Bioinformatics, The University of Texas Southwestern Medical Center, Dallas, TX 75390, USA; Department of Population and Data Sciences, The University of Texas Southwestern Medical Center, Dallas, TX 75390, USA; Lyda Hill Department of Bioinformatics, The University of Texas Southwestern Medical Center, Dallas, TX 75390, USA; Department of Population and Data Sciences, The University of Texas Southwestern Medical Center, Dallas, TX 75390, USA

**Keywords:** COVID-19, SARS-CoV-2, stochastic growth model, stochastic SIR model, time-series cross-validation

## Abstract

**Background:**

Forecasting of COVID-19 cases daily and weekly has been one of the challenges posed to governments and the health sector globally. To facilitate informed public health decisions, the concerned parties rely on short-term daily projections generated via predictive modeling. We calibrate stochastic variants of growth models and the standard susceptible-infectious-removed model into 1 Bayesian framework to evaluate and compare their short-term forecasts.

**Results:**

We implement rolling-origin cross-validation to compare the short-term forecasting performance of the stochastic epidemiological models and an autoregressive moving average model across 20 countries that had the most confirmed COVID-19 cases as of August 22, 2020.

**Conclusion:**

None of the models proved to be a gold standard across all regions, while all outperformed the autoregressive moving average model in terms of the accuracy of forecast and interpretability.

## Background

COVID-19, a respiratory disease caused by the coronavirus SARS-CoV-2, rapidly caused an ongoing global pandemic. By October 2020, COVID-19 had become the third leading cause of death in the USA for individuals aged 45–84 years, and it continues to spread quickly in most countries. Given the extent of health and economic distress caused by the pandemic, there is an urgent public health need to improve prediction of the spread of COVID-19 locally, nationally, and globally.

Since its emergence, a myriad of predictive modeling approaches have been proposed to forecast trends of COVID-19 disease to aid public health officials in developing effective policies and measures to suppress spread and minimize casualties. Five general approaches to forecast new cases or expected combined mortality linked to COVID-19 exist: (i) time-series forecasting such as autoregressive integrated moving average (ARIMA) [1,2], (ii) growth curve fitting based on the generalized Richards curve (GRC) or its special cases [3–7], (iii) compartmental modeling based on the susceptible-infectious-removed (SIR) models or their derivations [8–18], (iv) agent-based modeling [19], and (v) artificial intelligence (AI)-inspired modeling [20–23].

Each approach, whether deterministic or stochastic, has its own strengths. For example, the ARIMA model combines the regressive process and the moving average, allowing prediction of a given time series by considering its own lags and lagged forecast error. Curve-fitting approaches (also known as phenomenological modeling) fit a curve to the observed number of cumulative confirmed cases or deaths with a certain error structure (e.g., Gaussian or Poisson), enabling meaningful interpretation through curve parameters while accounting for measurement errors. Compartmental modeling (also known as mechanistic modeling) assigns partitions of the population to compartments corresponding to different stages of the disease and characterizes the disease transmission dynamics by the flow of individuals through compartments. Agent-based modeling approaches use computer simulations to study the dynamic interactions among the agents (e.g., people in epidemiology) and between an agent and the environment. AI-based modeling approaches usually combine time series, clustering, and forecasting, resulting in an exemplary predictive performance. Debate among researchers has grown over model performance evaluation and selection of the best model for a certain feature (e.g., cases, deaths), a particular regional level (e.g., county, state, country), and other parameters. Fair evaluation and comparison of the output of different forecasting methods have remained elusive [24] because models vary in their complexity and the variables and parameters that characterize the dynamic states of a system.

Although the literature has compared predictive models for infectious diseases, to our knowledge, existing work does not systematically compare their performance, specifically with the same amount of *a priori* available information. We calibrate stochastic variants of 6 different growth models (i.e., logistic, generalized logistic, Richards, generalized Richards, von Bertalanffy, and Gompertz) and the standard SIR model. All models can be included using an ordinary differential equation (ODE) into 1 flexible Bayesian modeling framework. We limited the analysis to these 2 modeling approaches because both not only produce good short- and long-term forecasts but also provide useful insights into the disease dynamics of COVID-19. The growth models provide an empirical approach without a specific theory on the mechanisms giving rise to the observed patterns in the cumulative infection data, while the compartmental models incorporate key mechanisms involved in the disease transmission dynamics that explain patterns in the observed data.

In our Bayesian modeling framework, the bottom level is represented by a negative binomial model that directly models infection count data and accounts for the over-dispersed observational errors. The top level is derived from a choice of growth or compartmental models that characterize certain disease transmission dynamics through ODE(s). The Markov chain Monte Carlo (MCMC) algorithm samples from the posterior distribution. The short-term forecasts derive from the resulting MCMC samples. We perform the rolling- origin cross-validation (ROCV) procedure to compare the prediction error of different stochastic models. We used the 20 countries with the most confirmed case numbers for a country-level analysis. Observations included that (i) as the models learned more, the predictive performance improved in general for all regions; (ii) none of the models proved to be a gold standard across all the regions; and (iii) the ARIMA model underperformed all stochastic models proposed in the article. We designed a graphical interface that allows users to interact with future COVID-19 trends at different geographic locations in the USA based on real-time COVID-19 data. This web portal is updated daily and used to inform local policy-makers and the general public (https://qiwei.shinyapps.io/PredictCOVID19/ with Biotools ID: bayesepimodels_webapp) (BayesEpiModels Web App, RRID:SCR_019292).

## Data Description

Let $\boldsymbol{C}=(C_1,\ldots ,C_T)$ be a sequence of cumulative confirmed case numbers observed at *T* successive equally spaced points in time (e.g., day) in a specific region, where each entry $C_t\in \mathbb {N}$ for *t* = 1, …, *T*. Further let *C*_0_ be the initial value and $\dot{\boldsymbol{C}}=(\dot{C}_1,\ldots ,\dot{C}_T)$ be the lag 1 difference of $\boldsymbol{C}$, where $\dot{C}_1=C_1-C_0$ and each following entry $\dot{C}_t=C_t-C_{t-1},t=2,\ldots ,T$, i.e., the difference between 2 adjacent observations. In the analysis and modeling of a series of reported infectious disease daily data, the time-series data could also be the cumulative death numbers, recovery case numbers, or their sums, denoted by $\boldsymbol{D}$ (Death), $\boldsymbol{E}$ (Recovery), and $\boldsymbol{R}$ (Removed), and their corresponding new case numbers, denoted by $\dot{\boldsymbol{D}}$, $\dot{\boldsymbol{E}}$, and $\dot{\boldsymbol{R}}$. Assuming a closed population with size *N*, the time-series data could also be the number of susceptible people, denoted by $\boldsymbol{S}$, with each entry *S_t_* = *N* − *C_t_*. In reality, only confirmed cases and deaths are reported in most regions. Recovery data are not available or are hindered by under-reporting issues if available. Thus, our main goal was to make predictions of the future trend of an infectious disease only based on the daily confirmed cases $\dot{\boldsymbol{C}}$.

## Analysis

In this section, we discuss the findings of our COVID-19 data analysis. We first implemented each of the growth models listed in Table 1 and the standard SIR model under the proposed Bayesian framework for the 20 countries with the most confirmed COVID-19 case numbers as of August 22, 2020. Input data were the sequence of daily confirmed cases $\dot{\boldsymbol{C}}$ only, which were accessible from the Johns Hopkins University Center for Systems Science and Engineering COVID-19 Data Repository (https://github.com/CSSEGISandData/COVID-19/). Several recent COVID-19 studies also based their analyses on this resource (see e.g., [25–27]). For our MCMC algorithms, we set 100,000 iterations with the first half as burn-in and chose weakly informative priors. We present numerical and graphical summaries for posterior inference and short-term forecasting. Our final goal was to compare the predictive performance of all models using ARIMA as a benchmark model.

**Table 1:** List of *g*( · )’s functions based on growth curves

Model	$g(C_{t-1},\boldsymbol{\Theta })$	Parameters $\boldsymbol{\Theta }$	Continuous curve *y*(*u*)	Value at the turning point	Examples
GRC	$\lambda C_{t-1}^p\left[1-\left(\frac{C_{t-1}}{K}\right)^\alpha \right]$	$K\in \mathbb {N}$ ,$\lambda \in \mathbb {R}^+$,*p* ∈ (0, 1),$\alpha \in \mathbb {R}^+$	NA	$\left(\frac{p}{p+\alpha }\right)^{1/\alpha }K$	[7,28,29]
Richards	$\lambda C_{t-1}\left[1-\left(\frac{C_{t-1}}{K}\right)^\alpha \right]$	$K\in \mathbb {N}$ ,$\lambda \in \mathbb {R}^+$,$\alpha \in \mathbb {R}+$	*K*[1 + *A*exp ( − λα*u*)]^−1/α^,where $A=-1+\left[\frac{K}{y(0)}\right]^\alpha$	$\left(\frac{1}{1+\alpha }\right)^{1/\alpha }K$	[30–34]
GLC	$\lambda C_{t-1}^p\left(1-\frac{C_{t-1}}{K}\right)$	$K\in \mathbb {N}$ ,$\lambda \in \mathbb {R}^+$,*p* ∈ (0, 1)	N/A	$\frac{p}{p+1}K$	[7,35,36,37]
Logistic	$\lambda C_{t-1}\left(1-\frac{C_{t-1}}{K}\right)$	$K\in \mathbb {N}$ ,λ ∈ (0, 1)	*K*[1 + *A*exp ( − λ*u*)]^−1^,where $A=-1+\frac{K}{y(0)}$	$\frac{1}{2}K$	[3,6,7,28,38]
von Bertalanffy	$\lambda C_{t-1}^\frac{2}{3}\left[1-\left(\frac{C_{t-1}}{K}\right)^\frac{1}{3}\right]$	$K\in \mathbb {N}$ ,$\lambda \in \mathbb {R}^+$	$K\left[1+A\exp (-\frac{1}{3}\gamma K^{-1/3} u)\right]^3$ ,where $A=1-\left[\frac{y(0)}{K}\right]^{1/3}$	$\frac{8}{27}K$	[6]
Gompertz	$\lambda C_{t-1}\log \frac{K}{C_{t-1}}$	$K\in \mathbb {N}$ ,λ ∈ (0, 1)	*K*exp [*A*exp ( − λ*u*)],where $A=\log \frac{y(0)}{K}$	$\frac{1}{e}K$	[6]
GGC	$\lambda C_{t-1}^p$	$\lambda \in \mathbb {R}^+$ ,*p* ∈ (0, 1)	[*A* + λ*u*(1 − *p*)]^1/(1 − *p*)^,where *A* = *y*(0)^1 − *p*^	NA	[7,29, 35, 36,39]

GGC: generalized growth curve; GLC: generalized logistic curve; GRC: generalized Richards curve; NA: not applicable.

### Forecasting of daily confirmed cases in the USA

**Figure 1:**
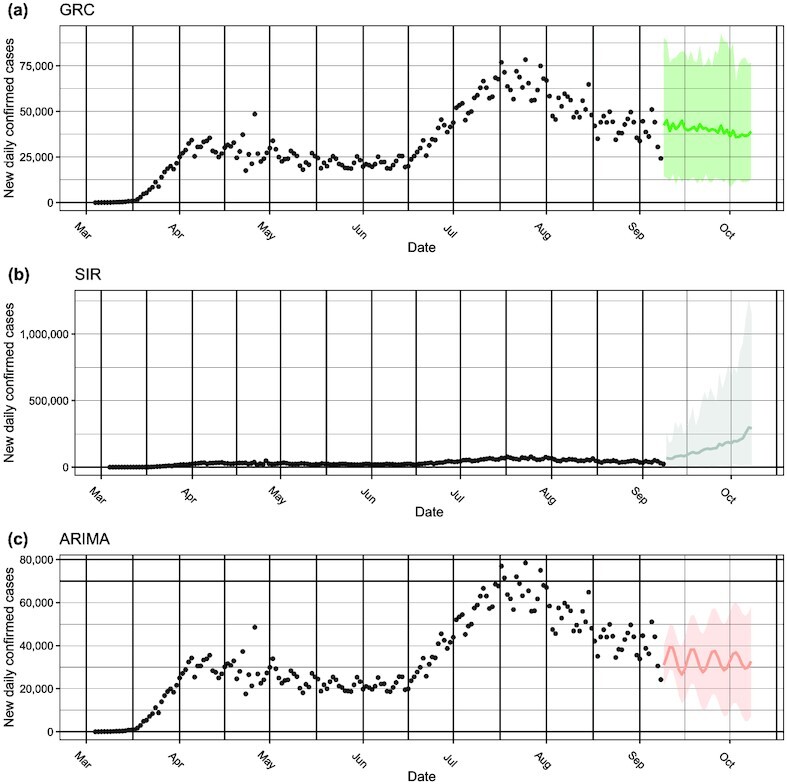
The 1-month forecasting of new daily confirmed COVID-19 cases in the USA made by the (a) GRC and (b) SIR model under the proposed Bayesian framework, as well as the benchmark (c) ARIMA model. The black circles represent the observed COVID-19 case numbers since early March 2020, while the colored circles and ribbons represent the predicted means and $95\%$ prediction intervals, respectively.

We first present the forecasting of U.S. daily confirmed cases made by the ARIMA model and our Bayesian framework with the choices of a GRC or SIR model. As seen in Figure 1, the GRC model demonstrates a downwards trend and the SIR model displays an upward trend, while the ARIMA model predicts a flat trajectory of daily predicted cases. A natural epidemiological interest is the estimated final size and end date of an epidemic. Growth models include a model parameter *K* that estimates the final epidemic size. For the SIR model, there is no available parameter that estimates the final size. Hence, the final case count is approximated as the predictive mean that converges to a specific value from the related MCMC samples. We applied a similar strategy to obtain the predicted mean of the final case counts using the ARIMA model [2]. The estimated cumulative confirmed cases by the end of 2020 were projected at 13.1, 106.1, and 10.0 (in millions), fitting the GRC, SIR, and ARIMA models, respectively. Assuming that the epidemic continues until the end of 2021, the final epidemic sizes are predicted to be 13.4, 187.3, and 22.0 (in millions) by the 3 models, respectively. To account for the discrepancies in forecasts and validate the forecast with actual reported figures, there is a need for an appropriate strategy to evaluate and compare the predictive performance of the concerned models.

### Model comparison through rolling-origin cross-validation

**Figure 2:**
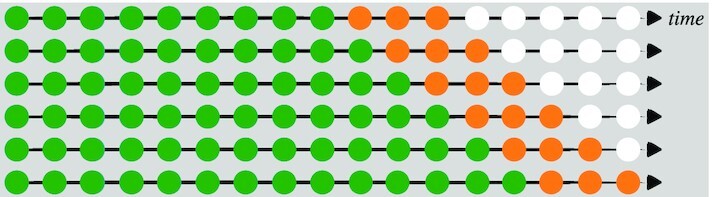
A visual guide to rolling-origin cross-validation (ROCV), where the total sample size *T* = 17, the initial training sample size is 9, and the testing sample size is 3. The green, orange, and white circles indicate training, testing, and unused samples in 1 CV iteration.

Cross-validation (CV) is a resampling procedure used to evaluate regression and classification models when only a limited data sample is available. The procedure randomly splits all data samples into 2 parts: training and testing sets, where the former is used to fit a model and the latter is used to evaluate the model’s prediction performance in terms of certain error measures. The key assumption of CV is that all data points should be independent and identically distributed (i.i.d.). Unfortunately, time-series data are serially auto-correlated, meaning that the observations are dependent on the time they were recorded. To circumvent this issue, the ROCV technique was proposed [40]. It splits the data into training and testing sets without affecting the i.i.d. assumption. We used an adaption of this method to evaluate the short-term forecasting performance among different top-level choices under the proposed Bayesian framework and ARIMA. Figure 2 shows the ROCV representation for an example of time-series data (*T* = 17). Algorithm 1 summarizes this evaluation procedure. The choice of initial training sample size (denoted by *k*) was set to 7 days to evaluate how well the models are able to generate forecasts during the initial phase of the pandemic, while the testing sample size (denoted by ω) was chosen to be 3 days to meet with our objective of comparing short-term forecasting performance. We defined the first day *t* = 1 as the date when cumulative confirmed case load per country reached 100, resulting in different days for different countries.

**Algorithm 1:**
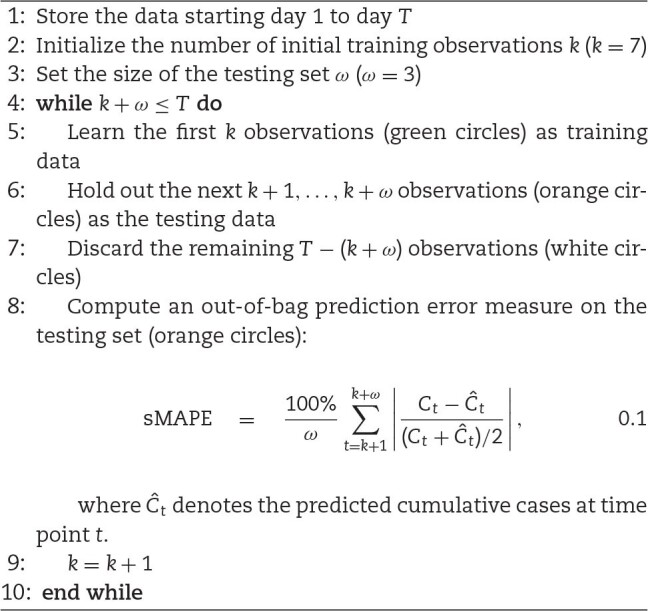
Rolling-origin cross-validation (ROCV)

A CV algorithm requires a predictive error metric that can quantify model performance in terms of forecasting accuracy. Root mean square error (RMSE) and mean absolute deviations (MAD) are candidates for error measures for out-of-bag predictions but are dependent on scale. Thus, large values may influence the errors to be larger. Mean absolute percentage error (MAPE) has been a widely used predictive measure owing to its interpretability and its independence from scale, although the distribution of such percentage errors can be skewed if the data consist of values close to zero. Moreover, there is a possibility of this measure being undefined due to a zero in the denominator. In addition, MAPE can be subjected to unbounded extreme values if the actual data points are close to zero or if the absolute forecasting error $(C_t - \hat{C}_t)$ is large. An improved percentage error metric, namely, symmetric mean absolute percentage error (sMAPE), was proposed to address these issues [40]. This measure bounded the error between 0% and 200% by incorporating the mean of actual and predicted cases $(C_t + \hat{C}_t)/2$ in the denominator. Values close to $0\%$ result from accurate predictions, while errors close to $200\%$ signify inaccurate forecasting. This metric was considered in our analysis as it addressed the problem of having an unbounded measure and provided better symmetry and interpretability compared to MAPE.

**Figure 3:**
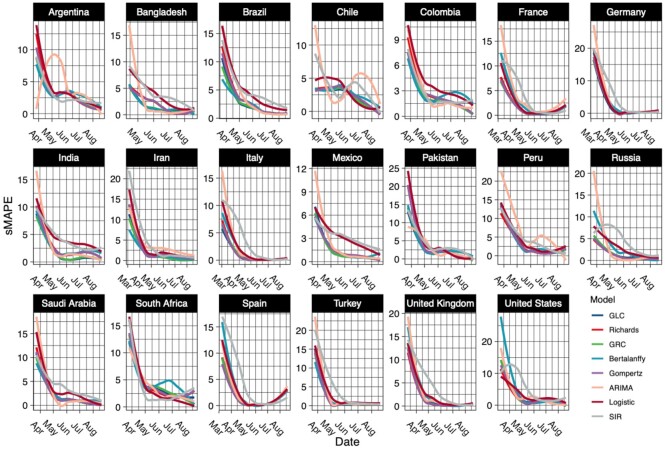
The smoothed sMAPE curves generated by the rolling-origin cross-validation (ROCV) over time for the 20 countries with the most confirmed COVID-19 case numbers as of August 22, 2020.

Figure 3 displays the smoothed sMAPE curves generated by the ROCV across time for the 20 countries with the most confirmed case numbers as of August 22, 2020. All models performed poorly in the early stage, but as more data became available to be learned, the predictive performance gradually improved as the sMAPE decreased. The ARIMA and SIR models were performing significantly worse than the growth models in the early phase, which may be attributable to ARIMA (not having the growth-specific parameters) being unable to detect the early growth. However, due to assumptions of a fixed transmission rate γ and under-reporting of data, SIR performed poorly. The stochastic growth curves were able to learn the epidemiological data trend in the initial phase with the help of the growth and scaling parameters. In the latter half of the epidemic, all the models were performing equally well. Hence, we were unable to conclude that any one particularly dominated the entire duration of the epidemic.

**Figure 4:**
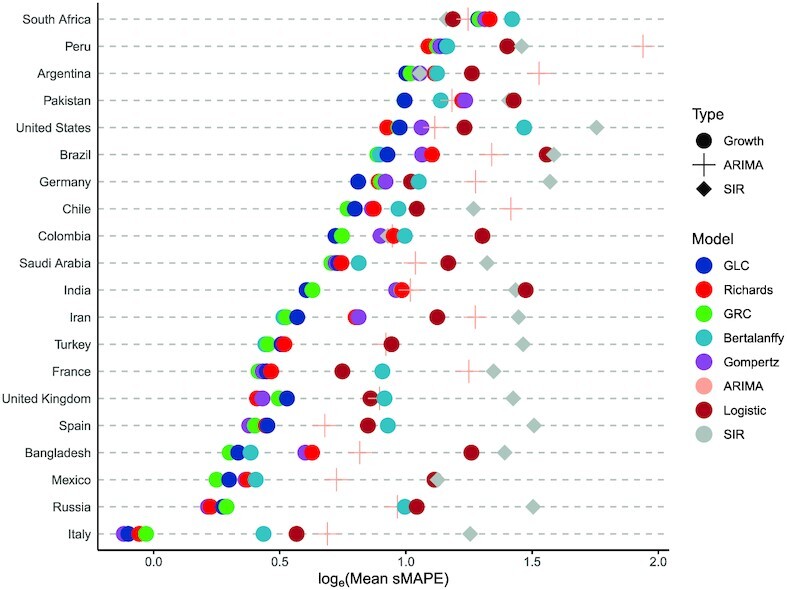
The Cleveland dot plot of the averaged sMAPE generated by the rolling-origin cross-validation (ROCV) for the 20 countries with the most confirmed COVID-19 case numbers as of August 22, 2020.

To answer the question whether we could pick 1 model with best predictive performance on average for any particular country, we constructed a Cleveland dot plot as shown in Figure 4 that allowed us to rank the model performance averaged over the entire pandemic by country. We arranged countries in ascending order of predictive performance.

## Discussion

We observed that all models performed best for Italy and worst for South Africa. The Richards model had the minimum averaged sMAPE for forecasting cumulative case counts in the USA, while the GRC model had the lowest averaged sMAPE across 7 countries followed by the GLC model with 4. The SIR model was the best performer for South Africa. The Richards, von Bertalanffy, and Gompertz models had a fair share of predictive dominance in the remaining countries. The ARIMA model performed below average across all countries.

The GRC and GLC models were consistent performers across all countries owing to their ability to detect subexponential growth rates at an early stage of an epidemic. The inclusion of the scale parameter α that could account for any asymmetry in the data allowed the GRC and Richards models to generally perform best in countries that did not have symmetric “S"-shaped growth patterns and displayed randomness as well as multiple peaks. Countries including the USA, Peru, Saudi Arabia, Iran, Turkey, and France displayed multiple peaks in the daily confirmed case counts. As a result, the Richards model performed the best in the USA, UK, and Peru, while the GRC model dominated in the remaining countries with multiple peaks.

We observed a random structure in countries like Brazil, Chile, Bangladesh, and Mexico. The GRC was the most complex model and performed the best in these countries. However, the GLC model usually performed better in countries that had a single peak and an approximate “S"-shaped curvature. The GLC model was able to generalize better than the GRC model when data were well structured and less random. Argentina, Pakistan, Germany, Colombia, and India had a single peak without much randomness, and the GLC model performed better in these countries. In South Africa, the usual growth models performed the worst owing to a staggering growth rate in the initial and the middle phase of the epidemic. The SIR model performed the best out of the worst while the logistic model performed well due to its simplicity. The Gompertz model was the best performer in Russia, Spain, and Italy because it generalizes better than the other models.

## Conclusion

We developed a number of stochastic variants of growth and compartmental models in a unified Bayesian framework. The literature has discussed a theoretical comparison of growth models in great detail [5–7, 28, 29, 35, 36, 38]. However, to our knowledge, no work systematically compares the performances among all as well as against a compartmental model such as the SIR model and a time-series forecasting model such as the ARIMA model.

We conclude that the proposed Bayesian framework not only allows room for interpretation but also offers an exemplary predictive performance when it comes to COVID-19 daily report data. Moreover, ARIMA (being a pure learning algorithm) is not able to match the forecasting accuracy of stochastic models. Furthermore, the model parameters of ARIMA do not provide any information of epidemiological interest.

In the future, we aim to develop an ensemble model that can aggregate the prediction of each base model, resulting in 1 final prediction for the unseen data. Note that a group of researchers have recently introduced a GGM-GLM ensemble model [35] and compared forecasting performance with the individual models for the Ebola Forecasting Challenge [41]. The ensemble model outperformed the others under some circumstances. We also plan to perform long-term forecasting evaluation using epidemic features described in [24]. A subepidemic wave model that could detect multiple peaks in the data has been recently developed [37] and has the potential to improve forecasting performance.

An “S”-shaped curvature on $\boldsymbol{C}$ attributes a simple growth model as it assumes that an epidemic would last only a short duration and that only a single peak would be observable on $\dot{\boldsymbol{C}}$. This oversimplified assumption could be problematic because COVID-19 is more likely to be an endemic. Moreover, the changing government policies and public health guidelines as well as population behaviors (holiday) led to variable disease transmission rates, resulting in multiple peaks. Thus, developing stochastic growth models with the addition of a change-point detection mechanism to account for multiple peaks is worth investigating. We have demonstrated that an approach that combines a change-point detection model and a stochastic SIR model could significantly improve the short-term forecasting of the new daily confirmed cases [42]. To handle the sophisticated extensions of the present work, we need to utilize advanced versions of the Metropolis-Hastings (MH) algorithm in the MCMC algorithms. For example, the MH with delayed rejection [43], the combination of delayed rejection and adaptive Metropolis samplers [44], the multiple-try Metropolis [45, 46], and the methods discussed in Liang et al. [47].

## Potential Implications

The proposed Bayesian epidemiological models in a unified framework lay the foundation for an integrative approach to model and predict epidemiological data with tremendous accuracy and interpretability. Growth and compartmental models obtained as solutions to ODEs are implemented to model epidemiological data under a deterministic setting as they provide a natural framework representative of such data types. However, the estimated model parameters crucial for providing insights into the nature of the epidemic are unreliable under the deterministic setting due to identifiability issues. The stochastic models mimic the structure of epidemiological models and incorporate parameter-specific priors and measurement errors to solve the issues. Researchers can follow a similar set-up to predict cases and deaths caused by an epidemic at any geographical level given the availability of data. Furthermore, the stochastic SIR model can be augmented by incorporating mobility, hospitalization, and recovery data, resulting in better forecasts. This work also promotes an algorithmic strategy to measure forecasting performances of time-series models in general.

On a much broader scale, this work encourages researchers to explore probabilistic approaches to model epidemiological data to develop computationally efficient algorithms that meet time and cost constraints.

## Methods

In this section, we present a bilevel Bayesian framework for predicting new confirmed cases during a pandemic in a closed society. The bottom level directly models the observed counts while accounting for measurement errors. Two alternatives for the top level are then introduced and characterize the epidemic dynamics through growth curve or compartmental trajectories, respectively. Before introducing the main components, we summarize the possibly observable data.

### Bottom level: time-series count-generating process

We consider that the new case numbers observed at time *t*, i.e., $\dot{C}_t$, are sampled from a negative binomial (NB) model,

\begin{eqnarray*}
\dot{C}_t\quad \sim \quad \text{NB}(g(C_{t-1}, \boldsymbol{\Theta }),\phi ),\quad t=2,\ldots ,T
\end{eqnarray*}

as it automatically accounts for measurement errors and uncertainties associated with the counts. Here, we use NB(μ, ϕ), μ, ϕ > 0 to denote an NB distribution with expectation μ and dispersion 1/ϕ. We assume that this stochastic process is a Markov process, where the present state (at time *t*) depends only upon its previous state (at time *t* − 1). Therefore, the NB mean is a function, denoted by *g*( · ), of the case number observed at time *t* − 1, characterized by a set of interpretable/uninterpretable model parameters $\boldsymbol{\Theta }$. With this parameterization, the NB variance is μ + μ^2^/ϕ, indicating that ϕ controls the variance of measurement error. A small value leads to a large variance to mean ratio, while a large value approaching infinity reduces the NB model to a Poisson model with the same mean and variance. We can write the full data likelihood as

(1) \begin{eqnarray*}
f(\boldsymbol{\dot{C}}|\boldsymbol{\Theta },\phi ) = \prod _{t=2}^T\frac{\Gamma (\dot{C}_t+\phi )}{\dot{C}_t!\Gamma (\phi )}\left[\frac{\phi }{g(C_{t-1}, \boldsymbol{\Theta })+\phi }\right]^\phi \left[\frac{g(C_{t-1}, \boldsymbol{\Theta })}{g(C_{t-1}, \boldsymbol{\Theta })+\phi }\right]^{\dot{C}_t}. \nonumber \\ \end{eqnarray*}

For the prior distribution of the dispersion parameter ϕ, we choose a gamma distribution, ϕ ∼ Ga(*a*_ϕ_, *b*_ϕ_). We recommend small values, such as *a*_ϕ_ = *b*_ϕ_ = 0.001, for a non-informative setting [48]. Note that the proposed framework can be viewed as a stochastic discrete-time state-space model with a negative binomial error structure. The proposed Bayesian models, on average, mimic the epidemic dynamics and are more flexible than those deterministic epidemiological models because they account for measurement error and have the potential to incorporate existing information into the prior structure of $\boldsymbol{\Theta }$.

### Top level I: Growth model

We first discuss the choices of *g*( · ) when implementing growth models. The development of a variety of growth curves originates from population dynamics [49] and growth of biological systems [50–53] modeling. A number of growth curves have been adapted in epidemiology for trend characterization and forecasting of an epidemic, such as the severe acute respiratory syndrome (SARS) [30,31], dengue fever [32,33], pandemic influenza A (H1N1) [34], Ebola virus disease [28,38], Zika fever [29], and COVID-19 [3,6,7,54].

The underlying assumption is that the rate of growth of a population, organism, or infectious individuals eventually declines with size. The logistic curve (also known as sigmoid curve) is the simplest growth curve of continuous time $u\in \mathbb {R}$. It is a non-negative symmetric “S”-shaped curve with equation $y(u)=K/\{1+\exp [-\lambda (u-u_0)]\}$, where *u*_0_ is the midpoint, *K* is the maximum value, and λ reflects the steepness of the curve. Clearly, *y*(*u*) approaches *K* when *u* → ∞, while it converges to zero when *u* → −∞. In fact, the continuous curve *y*(*u*) is the solution of a first-order non-linear ODE,

\begin{eqnarray*}
\frac{dy(u)}{du}\, =\, \lambda y(u)\left[1-\frac{y(u)}{K}\right]
\end{eqnarray*}

with condition *y*(*u*_0_) = *K*/2, where *dy*(*u*)/*du* can be interpreted as time-variant growth rate of the curve *y*. The above ODE reveals: (i) a non-negative growth rate, *dy*(*u*)/*du* > 0 as *y*(*u*) ∈ [0, *K*]; (ii) an approximately exponential growth at the initial stage, *y*(*u*) ≈ exp (λ*u*) as *dy*(*u*)/*du* ≈ λ*y*(*u*) when *y*(*u*) → 0; (iii) no growth at the final stage, *y*(*u*) *dy*(*u*)/*du* = 0 when *y*(*u*) → *K*; (iv) a maximum growth rate of λ*K*/4 occurred when *y*(*u*) = *K*/2, indicated by *d*^2^*y*(*u*)/*du*^2^ = λ*dy*(*u*)/*du*(1 − 2*y*(*u*)/*K*). Based on those curve characteristics, we can use the growth curve to characterize the trend of cumulative confirmed cases $\boldsymbol{C}$.

We mainly considered a family of growth curves that are derived from the GRC, which is the solution to the following ODE,

(2) \begin{eqnarray*}
\frac{dy(u)}{du} \, = \, \lambda y(u)^p\left\{1-\left[\frac{y(u)}{K}\right]^\alpha \right\}
\end{eqnarray*}

in continuous time *u*, while its discretized form at time point *t* is written as $y_{t}-y_{t-1}=\lambda y_{t-1}^p\left[1-\left({y_{t-1}}/{K}\right)^\alpha \right]$. For those model-specific parameters in the context of epidemiology, *K* is the final epidemic size and should be an integer in the range of (0, *N*], where *N* is the total population, $\lambda \in \mathbb {R}^+$ is the infectious rate at early epidemic stage, *p* ∈ (0, 1) is known as scaling of growth, and $\alpha \in \mathbb {R}^+$ controls the curve symmetry. As our observed infectious disease data are usually counts collected at successive equally spaced discrete time points, we formulate the NB mean function *g*( · ) based on the discretized form of (2),

(3) \begin{eqnarray*}
g(C_{t-1},\boldsymbol{\Theta }=\lbrace K,\lambda ,p,\alpha \rbrace )\quad =\quad \lambda C_{t-1}^p\left[1-\left(\frac{C_{t-1}}{K}\right)^\alpha \right]. \end{eqnarray*}

Table 1 provides a list of *g*( · )’s for growth curves with their characteristics. All the listed growth curves have been utilized and discussed in previous epidemiological studies. We include all of those choices in our framework excluding the last one, which is based on the generalized growth curve (GGC), because it lacks the final epidemic size *K* specification.

Without any existing information, we assume that *K* is from a discrete uniform distribution in its range and γ is from a γ- or a β-distribution, depending on the choice of growth curves. For example, for both logistic and Gompertz curves, we assume γ ∼ β(*a*_γ_, *b*_γ_), a natural modeling choice for parameter value restricted to the (0,1) interval, and suggest choosing *a*_γ_ = *b*_γ_ = 1 for a uniform setting; otherwise, we place a γ prior, i.e., γ ∼ Ga(*a*_γ_ = 0.001, *b*_γ_ = 0.001). For the choice of GRC and generalized logistic curve (GLC), the prior of *p* is chosen to be β(*a_p_* = 1, *b_p_* = 1). Last, we set α ∼ Ga(*a*_γ_ = 0.001, *b*_γ_ = 0.001) for fitting a GRC or Richards curve.

### Top level II: Compartmental model

The SIR model was developed to simplify the mathematical modeling of human-to-human infectious diseases by Kermack and McKendrick [55]. It is a fundamental compartmental model in epidemiology. At any given time *u*, each individual in a closed population with size *N* is assigned to 3 distinctive compartments with labels: susceptible (*S*), infectious (*I*), or removed (*R*, being either recovered or dead). The standard SIR model describes the flow of people from *S* to *I* and then from *I* to *R* by the following set of nonlinear ODEs:

\begin{eqnarray*}
\left\lbrace \begin{array}{@{}l@{\quad }l@{}}\begin{array}{l}\frac{dS(u)}{du}\quad =\quad -\beta S(u)\frac{I(u)}{N}\\ \frac{dI(u)}{du}\quad =\quad \beta S(u)\frac{I(u)}{N}-\gamma I(u)\\ \frac{dR(u)}{du}\quad =\quad \gamma I(u)\\ \end{array} \end{array}\right., \end{eqnarray*}

where *S*(*u*), *I*(*u*), and *R*(*u*) are the population numbers of susceptible, infectious, and removed compartments measured in time *u*, subject to *S*(*u*) + *I*(*u*) + *R*(*u*) = *N*, ∀*u*. Another nature constraint is *dS*(*u*)/*du* + *dI*(*u*)/*du* + *dR*(*u*)/*du* = 0. Here, $\beta \in \mathbb {R}^+$ is the disease transmission rate, $\gamma \in \mathbb {R}^+$ is the removal rate, and their ratio $\mathcal {R}_0=\beta /\gamma$ is defined as the “basic reproduction number." The rationale behind the first equation is that the number of new infections during an infinitesimal amount of time, −*dS*(*u*)/*du*, is equal to the number of susceptible people, *S*(*u*), times the product of the contact rate, *I*(*t*)/*N*, and the disease transmission rate β. The third equation reflects that the infectious individuals leave the infectious population per unit time, *dI*(*u*)/*du*, at a rate of γ*I*(*u*). The second equation follows immediately from the first and third equations as a result of *dS*(*u*)/*du* + *dI*(*u*)/*du* + *dR*(*u*)/*du* = 0. Assuming that only a small fraction of the population is infected or removed in the onset phase of an epidemic, we have *S*(*u*)/*N* ≈ 1 and thus the second equation reduces to *dI*(*u*)/*du* = (β − γ)*I*(*u*), revealing that the infectious population is growing if and only if β > γ. As the expected lifetime of an infected case is given by γ^−1^, the ratio $\mathcal {R}_0=\beta /\gamma$ is the average number of new infectious cases directly produced by an infected case in a completely susceptible population. The so-called basic reproduction number is a good indicator of an infectious disease’s transmissibility.

We only consider the standard SIR model, although it is still feasible to design *g*( · )’s from its variations (see a comprehensive summary [56]), such as the susceptible-infectious (SIS) model, the susceptible-infectious-recovered-deceased (SIRD) model, the susceptible-exposed-infectious-removed (SEIR) model, the susceptible-exposed-infectious-susceptible (SEIS) model, and their versions with a maternally derived immunity compartment [57], as well as the recently developed extended-SIR (eSIR) model [14]. For modeling discrete time-series data, we use the discrete-time version of the standard SIR model,

(4) \begin{eqnarray*}
\left\lbrace \begin{array}{@{}l@{\quad }l@{}}\begin{array}{l}\dot{S}_{t} = -\beta S_{t-1}\frac{I_{t-1}}{N}\\ \dot{I}_{t} = \beta S_{t-1}\frac{I_{t-1}}{N}-\gamma I_{t-1}\\ \dot{R}_{t} = \gamma I_{t-1}\\ \end{array} \end{array}\right., \end{eqnarray*}

where $\dot{S}_{t}=S_t-S_{t-1}$, $\dot{I}_{t}=I_t-I_{t-1}$, and $\dot{R}_{t}=R_t-R_{t-1}$ are the differences between 2 adjacent observations in the sequence of susceptible, infectious, and removed case numbers, respectively. The model has 3 trajectories, 1 for each compartment. The compositional nature of the 3 trajectories implies that we only need 2 of the 3 sequence data, e.g., *S_t_* = *N* − *C_t_* and *R_t_* for *t* = 1, …, *T*. However, recovery data only exist in a few regions and are hindered by under-reporting even if they exist, which makes both model inference and predictions infeasible. Alternatively, we consider both the removed and actively infectious cases as missing data and mimic their relationship in spirit to some compartmental models in epidemiology. Specifically, we assume that the number of new removed cases at time *t*, i.e., $\dot{R}_t$, is sampled from a Poisson distribution with mean γ*I*_*t* − 1_, i.e., $\dot{R}_t\sim \text{Poi}(\gamma I_{t-1})=\text{Poi}(\gamma (N-C_{t-1}-R_{t-1}))$, where γ should be specified. Such a strategy but with different error structure was also considered in some other compartmental models in epidemiology [16,58,59]. We can estimate the value of γ from publicly available high-quality data where confirmed cases, deaths, and recovered cases are all well documented, or from prior epidemic studies due to the same under-reporting issue in actual data. In this article, we choose the removal rate γ = 0.1 as suggested by Weitz et al. [60]. Based on this simplification, we rewrite the first equation in (4) as,

\begin{eqnarray*} (N-C_t)-(N-C_{t-1}) = -\beta (N-C_{t-1})\frac{N-C_{t-1}-R_{t-1}}{N}, \end{eqnarray*}

resulting in

\begin{eqnarray*}
\dot{C}_t = \beta (N-C_{t-1})\frac{N-C_{t-1}-R_{t-1}}{N}. \end{eqnarray*}

Thus, we formulate the NB mean function *g*( · ) for the standard SIR model as,

(5) \begin{eqnarray*}
g(C_{t-1},{\boldsymbol{\Theta }} = \lbrace \beta \rbrace |{\boldsymbol{R}}) = \beta ({N-C}_{t-1})\frac{{N-C}_{t-1}-R_{t-1}}{N}, \end{eqnarray*}

where $\boldsymbol{R}$ can be sequentially inferred from $\boldsymbol{C}$.

Without any existing information, in our Bayesian framework we assume β from a γ-distribution with hyperparameters that makes both the mean and variance of the transformed variable $\mathcal {R}_0=\beta /\gamma$ equal to 1, i.e., β ∼ Ga(1, 1/γ).

## Model Fitting

In this section, we briefly describe the MCMC algorithm for posterior inference and forecasting. Our Bayesian inferential strategy allows us to simultaneously infer all model-specific parameters and quantify their uncertainties.

### MCMC algorithms

We first describe how to update the dispersion parameter ϕ in the proposed Bayesian framework because it does not depend on the choice of models. At each MCMC iteration, we perform the following step:

Update of dispersion parameter ϕ. We update ϕ by using a random walk Metropolis-Hastings (RWMH) algorithm. We first propose a new ϕ*, of which logarithmic value is generated from $\text{N}\left(\log \phi ,\tau _\phi ^2\right)$ and then accept the proposed value ϕ* with probability min (1, *m*_MH_), where the Hastings ratio is

\begin{eqnarray*}
m_{\rm MH} = \frac{f({\boldsymbol{\dot{C}}} | {\phi} ^{*},\boldsymbol{\Theta })}{f(\boldsymbol{\dot{C}}|\phi ,\boldsymbol{\Theta })}\frac{\pi \left({\phi }^{*}\right)}{\pi \left({\phi }\right)}\frac{J\left({\phi }\leftarrow {\phi }^{*}\right)}{J\left({{\phi }^{*}\leftarrow \phi }\right)}. \end{eqnarray*}

Here we use *J*( · ← · ) to denote the proposal probability distribution for the selected move. Note that the last term, which is the proposal density ratio, cancels out for this RWMH update.

#### Top level as a growth model

We only present the updates of each parameter in the GRC model because all other derivative models are its special cases. Our primary interest lies in the estimation of the final pandemic size *K* and the infectious rate at early epidemic stage λ.

Update of final epidemic size parameter *K*. We update *K* by using an RWMH algorithm. We first propose a new K*, of which logarithmic value is generated from a truncated Poisson distribution Poi(log *K*) within [log *C_T_*, log *N*], and then accept the proposed value K* with probability min (1, *m*_MH_), where the Hastings ratio is

\begin{eqnarray*}
m_{\rm MH} = \frac{f(\boldsymbol{\dot{C}}|{\phi} ,{K}^{*},\lambda ,p,{\alpha} )}{f(\boldsymbol{\dot{C}}|{\phi} ,{K},\lambda ,p,\alpha )}\frac{\pi \left({K}^{*}\right)}{\pi \left({K}\right)}\frac{J\left({K}\leftarrow {K}^{*}\right)}{J\left({{K}^{*}\leftarrow {K}}\right)}. \end{eqnarray*}

Note that with a discrete uniform prior on $\boldsymbol{K}$, the last 2 terms cancel out for this RWMH update.

Update of infectious rate parameter λ. We update λ by using an RWMH algorithm. We first propose a new λ*, of which logarithmic value is generated from $\text{N}\left(\log \lambda ,\tau _\lambda ^2\right)$, and then accept the proposed value λ* with probability min (1, *m*_MH_), where the Hastings ratio is

\begin{eqnarray*}
m_{\rm MH} = \frac{f(\boldsymbol{\dot{C}}|{\phi} ,K,{\lambda} ^{*},p,\alpha )}{f(\boldsymbol{\dot{C}}|{\phi} ,K,\lambda ,p,{\alpha} )}\frac{\pi \left({\lambda }^{*}\right)}{\pi \left({\lambda }\right)}\frac{J\left({\lambda }\leftarrow {\lambda }^{*}\right)}{J\left({{\lambda }^{*}\leftarrow \lambda }\right)}. \end{eqnarray*}

Note that the last term, which is the proposal density ratio, cancels out for this RWMH update.

**U**pdate of growth scaling parameter *p*. We update *p* by using an RWMH algorithm. We first propose a new p*, of which logarithmic value is generated from a truncated normal distribution $\text{N}\left(\log p,\tau _p^2\right)$ within [ − ∞, 0], and then accept the proposed value p* with probability min (1, *m*_MH_), where the Hastings ratio is

\begin{eqnarray*}
m_{\rm MH} = \frac{f(\boldsymbol{\dot{C}}|{\phi} ,K,\lambda ,{p}^{*},{\alpha} )}{f(\boldsymbol{\dot{C}}|{\phi} ,K,\lambda ,p,{\alpha} )}\frac{\pi \left({p}^{*}\right)}{\pi \left({p}\right)}\frac{J\left({p}\leftarrow {p}^{*}\right)}{J\left({{p}^{*}\leftarrow {p}}\right)}. \end{eqnarray*}

Note that with a uniform prior on *p* over its range [0,1], the last 2 terms cancel out for this RWMH update.

Update of symmetry parameter α. We update α by using an RWMH algorithm. We first propose a new α*, of which logarithmic value is generated from $\text{N}\left(\log \alpha ,\tau _\alpha ^2\right)$, and then accept the proposed value α* with probability min (1, *m*_MH_), where the Hastings ratio is

\begin{eqnarray*}
m_{\rm MH} = \frac{f(\boldsymbol{\dot{C}}|{\phi} ,K,\lambda ,p,{\alpha} ^{*})}{f(\boldsymbol{\dot{C}}|{\phi} ,K,{\lambda} ,p,{\alpha} )}\frac{\pi \left({\alpha }^{*}\right)}{\pi \left({\alpha }\right)}\frac{J\left({\alpha }\leftarrow {\alpha }^{*}\right)}{J\left({{\alpha }^{*}\leftarrow {\alpha} }\right)}. \end{eqnarray*}

Note that the last term, which is the proposal density ratio, cancels out for this RWMH update.

#### Top level as a compartmental model

Our primary interest lies in the estimation of the reproduction number $\mathcal {R}_0=\beta /\gamma$, which reflects the transmissibility of the disease. With our assumption that γ is given, we propose the following updates in each MCMC iteration.

Generate $\boldsymbol{R}$ based on $\boldsymbol{C}$. We assume *I*_1_ = *C*_1_, i.e., all the confirmed cases are capable of passing the disease to all susceptible individuals in a closed population at the very beginning. In other words, *R*_1_ = 0. Then we sample $\dot{R_2}\sim \text{Poi}(\gamma I_1)$, where γ is a prespecified tuning parameter and $\dot{R}_2=R_2-R_1$ ($\dot{R}_2=R_2$ here in that *R*_1_ = 0) is the new removed case numbers at time *t* = 2. Owing to the compositional nature, we can compute $I_2=I_1+\dot{C}_2-\dot{R}_2$, where $\dot{C}_2=C_2-C_1$ is the new confirmed case numbers at time *t* = 2. Next, we repeat this process of sampling $\dot{R}_t\sim \text{Poi}(\gamma I_{t-1})$ and computing $I_t=I_{t-1}+\dot{C}_t-\dot{R}_t,t=3,\ldots ,T$, to generate the sequence $\boldsymbol{R}$.

Update of reproduction number parameter β. We update β by using an RWMH algorithm. We first propose a new β*, of which logarithmic value is generated from a truncated normal distribution $\text{N}\left(\log \beta ,\tau _{\beta }^2\right)$, and then accept the proposed value β* with probability min (1, *m*_MH_), where the Hastings ratio is

\begin{eqnarray*}
m_{\rm MH} = \frac{f(\boldsymbol{\dot{C}}|{\beta} ^{*},\boldsymbol{R})}{f(\boldsymbol{\dot{C}}|{\beta} ,\boldsymbol{R})}\frac{\pi \left({\beta} ^{*}\right)}{\pi \left({\beta }\right)}\frac{J\left({\beta }\leftarrow {\beta }^{*}\right)}{J\left({{\beta} ^{*}\leftarrow \beta }\right)}. \end{eqnarray*}

Note that the last term, which is the proposal density ratio, cancels out for this RWMH update.

### Posterior inference

We obtain posterior inference by post-processing the MCMC samples Update of reproduction number parameterafter burn-in. Suppose that a sequence of MCMC samples on θ, θ ∈ {ϕ, *K*, λ, *p*, α, β},

\begin{eqnarray*}
\theta ^{(1)},\ldots ,\theta ^{(U)}
\end{eqnarray*}

has been collected, where *u, u* = 1, …, *U* indexes the iteration after burn-in. An approximate Bayesian estimator of each parameter can be obtained simply by averaging over the samples, $\hat{\theta }=\sum _{u=1}^U{\theta }^{(u)}/U$. In addition to that, a quantile estimation or credible interval for each parameter of interest can be obtained from this sequence as well.

### Forecasting

On the basis of the sequences of MCMC samples on *K*, λ, *p*, and α in the growth model or β in the compartmental model, we can predict the cumulative or new confirmed cases at any future time *T_f_* by Monte Carlo simulation. Particularly, from time *T* + 1 to *T_f_*, we sequentially generate

(6) \begin{equation*}
\dot{C}_t^{(u)}\quad \sim \quad \text{NB}(g(C_{t-1}, \boldsymbol{\Theta }^{(u)}),\phi ^{(u)}),\quad t=T+1,\ldots ,T_f. \end{equation*}

Then, both short- and long-term forecast can be made by summarizing the (*T_f_* − *T*)-by-*U* matrix of MCMC samples. For example, the mean predictive number of cumulative and new confirmed cases at time *T* + 1 are $\sum _{u=1}^U{C}_{T+1}^{(u)}/U$ and $\sum _{u=1}^U\dot{C}_{T+1}^{(u)}/U$, respectively.

## Software

**Figure 5:**
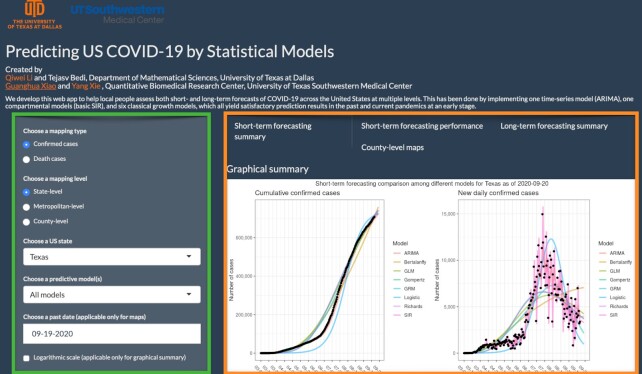
The web interface of the COVID-19 trend analysis page. The green box highlights the input panel that allows users to choose different mapping types and levels for a region. The orange box highlights the visualizations for short-term forecasting as per the instructions of users. Other tabs offer different graphs for summarizing the model performance, long-term forecasting, and county-level spatial maps.

This article introduces a user-friendly interactive web application (https://qiwei.shinyapps.io/PredictCOVID19/ with Biotools ID: bayesepimodels_webapp) (BayesEpiModels Web App, RRID:SCR_019292) integrated with the R Shiny package. Shiny is a web platform that allows users to interact with real-time data and use a myriad of visualization tools to analyze them. Figure 5 shows a screenshot of the web application. The web application has been developed to help the general public assess both short- and long-term forecasts of COVID-19 across the USA at both state and metropolitan level. The numbers of cumulative or new daily confirmed cases as well as deaths are projected by different growth models and the SIR model under the proposed Bayesian framework. Alongside the numerical summaries, users can view and interpret the trends that cover the same information. To validate the short-term forecasting, numerical and graphical summaries of MAE and MAPE of the predictions are provided for the more advanced users. Moving on to the long-term forecasting, the models estimate the peak number of cases and deaths, as well as their respective dates. Moreover, predictive estimates for the final size and date are also offered. Finally, for the users keen on visualizing the currently observed cases at a geographical level, the website offers county-level spatial maps.

## Availability of Source Code and Requirements

- Project name: BayesEpiModels
- Project home page: https://github.com/liqiwei2000/BayesEpiModels
- Operating systems: Windows and Linux
- Programming language: R (version 3.6.0)
- Other requirements: None
- License: GNU General Public License v3.0
- Biotools ID: bayesepimodels
- RRID:SCR_019291

## Data Availability

The related R/C++ codes for model preparation and execution are available on GitHub at https://github.com/liqiwei2000/BayesEpiModels,with snapshots in the *GigaScience* GigaDB repository [61]. The R Shiny web application is available for users at https://qiwei.shinyapps.io/PredictCOVID19/. The COVID-19 data repository is operated by the Johns Hopkins University Center for Systems Science and Engineering (JHU CSSE) and is freely available on GitHub at https://github.com/CSSEGISandData/COVID-19/.

## Abbreviations

AI: artificial intelligence; ARIMA: autoregressive integrated moving average; COVID-19: coronavirus disease–2019; CV: cross-validation; eSIR: extended susceptible-infectious-removed; GGC: generalized growth curve; GGM-GLM: generalized growth model–generalized logistic model; GLC: generalized logistic curve; GRC: generalized Richards curve; i.i.d.: independent and identically distributed; JHU CSSE: Johns Hopkins University Center for Systems Science and Engineering; MAD: mean absolute deviations; MAPE: mean absolute percentage error; MCMC: Markov chain Monte Carlo; NB: negative binomial; ODE: ordinary differential equation; RMSE: root mean square error; ROCV: rolling-origin cross-validation; RWMH: random walk Metropolis-Hastings; SARS-Cov-2: severe acute respiratory syndrome coronavirus 2; SEIR: susceptible-exposed-infectious-removed; SEIS: susceptible-exposed-infectious-susceptible; SIR: susceptible-infectious-removed; SIRD: susceptible-infectious-recovered-deceased; SIS: susceptible-infectious-susceptible; sMAPE: symmetric mean absolute percentage error.

## Competing Interests

The authors declare that they have no competing interests.

## Funding

This work was supported by the University of Texas at Dallas (UT Dallas) Office of Research (UT Dallas Center for Disease Dynamics and Statistics) and partially supported by the National Institutes of Health (1R01GM115473, 1R01GM140012, 5R01CA152301, P30CA142543, P50CA70907, R35GM136375); and the Cancer Prevention and Research Institute of Texas (RP180805, RP190107).

## Authors' Contributions

Q.L. developed the Bayesian framework, designed the MCMC algorithms, and constructed the R Shiny web application. Q.L. and T.B. contributed to the review of different methods and collaborated in the real data analysis. Q.L., C.U.L., G.X., and Y.X. conceived the study and supervised the web application development and the statistical analyses. All authors contributed to the writing of the manuscript. All authors have read and approved the final manuscript.

## Supplementary Material

giab009_GIGA-D-20-00328_Original_Submission

giab009_GIGA-D-20-00328_Revision_1

giab009_GIGA-D-20-00328_Revision_2

giab009_Response_to_Reviewer_Comments_Original_Submission

giab009_Response_to_Reviewer_Comments_Revision_1

giab009_Reviewer_1_Report_Original_Submission Luca Martino -- 11/26/2020 Reviewed

giab009_Reviewer_2_Report_Original_Submission Vasileios Basios -- 12/16/2020 Reviewed

giab009_Supplemental_File
