# Supplementary material for: Evaluating short-term forecasting of COVID-19 cases among different epidemiological models under a Bayesian framework
Source: Gigascience. 2021 Feb 19;10(2):giab009. doi: 10.1093/gigascience/giab009 (PMC7928884; doi:10.1093/gigascience/giab009)
Supplement: giab009_GIGA-D-20-00328_Original_Submission [file giab009_giga-d-20-00328_original_submission.pdf]

GigaScience

Evaluating Short-term Forecasting among Different Epidemiological Models under a Bayesian Framework

--Manuscript Draft--

**Manuscript Number:**	GIGA-D-20-00328
**Full Title:**	Evaluating Short-term Forecasting among Different Epidemiological Models under a Bayesian Framework
**Article Type:**	Research
**Funding Information:**	
**Abstract:**	Forecasting of COVID-19 daily confirmed cases has been one of the several challenges posed to the governments and health sectors on a global scale. To facilitate informed public health decisions, the concerned parties rely on short-term daily projections generated via predictive modeling. We calibrate stochastic variants of growth models and the standard SIR model into one Bayesian framework to evaluate their short-term forecasts. We implement rolling-origin cross-validation to compare the short-term forecasting performance of the stochastic epidemiological models and the autoregressive moving average model across the top 20 countries with the most confirmed cases as of August 22, 2020. In summary, it was noted that none of the models proved to be golden standards across all the regions in their entirety, while all outperformed the autoregressive moving average model in terms of the accuracy of forecast and interpretability.
**Corresponding Author:**	Qiwei Li, Ph.D.<br>University of Texas at Dallas<br>Richardson, TX UNITED STATES
**Corresponding Author Secondary Information:**	
**Corresponding Author's Institution:**	University of Texas at Dallas
**Corresponding Author's Secondary Institution:**	
**First Author:**	Qiwei Li, Ph.D.
**First Author Secondary Information:**	
**Order of Authors:**	Qiwei Li, Ph.D.
	Tejasv Bedi
	Christoph U. Lehmann, M.D.
	Guanghua Xiao, Ph.D.
	Yang Xie, Ph.D., M.D.
**Order of Authors Secondary Information:**	
**Additional Information:**	
**Question**	**Response**
Are you submitting this manuscript to a special series or article collection?	No
**Experimental design and statistics**<br><br>Full details of the experimental design and statistical methods used should be given in the Methods section, as detailed in our	Yes

Powered by Editorial Manager® and ProduXion Manager® from Aries Systems Corporation

Minimum Standards Reporting Checklist. Information essential to interpreting the data presented should be made available in the figure legends.<br><br>Have you included all the information requested in your manuscript?	
**Resources**<br><br>A description of all resources used, including antibodies, cell lines, animals and software tools, with enough information to allow them to be uniquely identified, should be included in the Methods section. Authors are strongly encouraged to cite Research Resource Identifiers (RRIDs) for antibodies, model organisms and tools, where possible.<br><br>Have you included the information requested as detailed in our Minimum Standards Reporting Checklist?	Yes
**Availability of data and materials**<br><br>All datasets and code on which the conclusions of the paper rely must be either included in your submission or deposited in publicly available repositories (where available and ethically appropriate), referencing such data using a unique identifier in the references and in the "Availability of Data and Materials" section of your manuscript.<br><br>Have you have met the above requirement as detailed in our Minimum Standards Reporting Checklist?	Yes

LaTeX - Main Document (TeX file)

Click here to access/download;LaTeX - Main Document (TeX file);main.tex

GigaScience, 2017, 1–11

doi: xx.xxxx/xxxx
Manuscript in Preparation
Paper

PAPER

Evaluating Short-term Forecasting among Different Epidemiological Models under a Bayesian Framework

Qiwei Li[1,†,*], Tejasv Bedi[1,†], Christoph U. Lehmann[2], Guanghua Xiao[2] and Yang Xie[2]

[1]The University of Texas at Dallas, Richardson, TX 75080, USA and [2]The University of Texas Southwestern Medical Center, Dallas, TX 75390, USA

*qiwei.li@utdallas.edu
†Contributed equally.

Abstract

Background: Forecasting of COVID-19 daily confirmed cases has been one of the several challenges posed to the governments and health sectors on a global scale. To facilitate informed public health decisions, the concerned parties rely on short-term daily projections generated via predictive modeling. We calibrate stochastic variants of growth models and the standard SIR model into one Bayesian framework to evaluate their short-term forecasts. **Results:** We implement rolling-origin cross-validation to compare the short-term forecasting performance of the stochastic epidemiological models and the autoregressive moving average model across the top 20 countries with the most confirmed cases as of August 22, 2020. **Conclusion:** In summary, it was noted that none of the models proved to be golden standards across all the regions in their entirety, while all outperformed the autoregressive moving average model in terms of the accuracy of forecast and interpretability.

Key words: COVID-19; SARS-CoV-2; Stochastic growth model; Stochastic SIR model; Time-series cross-validation.

Background

COVID-19, a respiratory disease coronavirus SARS-CoV-2, has rapidly created an ongoing global pandemic. It has become one of the leading causes of deaths in the United States (U.S.) and continues to spread fast in most other countries. Given the extent of the physical and economic suffering caused by the pandemic, there is an urgent public health need to better predict the spread of COVID-19 locally, nationally and globally. Since the emergence of the COVID-19 outbreak, a myriad of predictive modeling approaches have been proposed to predict trends of the disease to allow public health officials to develop effective policies and measures to suppress spread and minimize casualties. The five general approaches to forecast the number of new cases or the expected total mortality caused by the COVID-19 exist: 1) time-series forecasting such as autoregressive integrated moving average (ARIMA) [1, 2]; 2) growth curve fitting based on the generalized Richards curve (GRC) or its special cases [3, 4, 5, 6, 7]; 3) compartmental modeling based on the susceptible-infectious-removed (SIR) models or their derivations [8, 9, 10, 11, 12, 13, 14, 15, 16, 17, 18]; 4) agent-based modeling [19]; 5) artificial intelligence (AI)-inspired modeling [20, 21, 22, 23].

Each approach, whether deterministic or stochastic, has its own strengths. For instance, the ARIMA model combines the regressive process and the moving average, allowing to predict a given time series by considering its own lags and lagged forecast error. Curve fitting approaches (also known as phenomenological modeling) fit a curve to the observed number of cumulative confirmed cases or deaths with a certain error struc-

Compiled on: November 9, 2020.
Draft manuscript prepared by the author.

ture (e.g. Gaussian or Poisson), enabling meaningful interpretation through curve parameters while accounting for measurement errors. Compartmental modeling (also known as mechanistic modeling) approaches assign partitions of the population to compartments corresponding to different stages of the disease and characterize the disease transmission dynamics by the flow of individuals through compartments. Agent-based modeling approaches use computer simulations to study the dynamic interactions among the agents (e.g. people in epidemiology) and between an agent and the environment. AI-based modeling approaches usually combines time series, clustering, and forecasting, resulting in an exemplary predictive performance. There has been a growing debate among researchers over model performance evaluation and how to find the best model appropriate for a certain feature (cases, deaths, etc.), a particular regional level (county, state, country, etc.), and other parameters. Fair evaluation and comparison of the output of different forecasting methods have remained elusive [24] since models vary in their complexity and the number of variables and parameters that characterize the dynamic states of a system.

Although predictive models for infectious diseases have been compared in the literature, to our best knowledge, existing work does not systematically compare performances, specifically with the same amount of available information. We calibrate stochastic variants of six different growth models (i.e. logistic, generalized logistic, Richards, generalized Richards, Bertalanffy, and Gompertz) and the standard SIR model, all of which can be specified by an ordinary differential equation(ODE), into one flexible Bayesian modeling framework. The main reason for limiting our analysis to these two modeling approaches is that both not only produce good short and long-term forecasts, but also provide useful insights to understand the disease dynamics of COVID-19. The growth models provide an empirical approach without a specific theory on the mechanisms that give rise to the observed patterns in the cumulative infection data, while the compartmental models incorporate key mechanisms involved in the disease transmission dynamics to explain patterns in the observed data.

In our Bayesian modeling framework, the bottom-level is represented by a negative binomial model that directly models infection count data and accounts for the over-dispersed observational errors. The top-level is derived from a choice of growth or compartmental models that characterizes a certain disease transmission dynamic through ODE(s). The Markov chain Monte Carlo (MCMC) algorithm is used to sample from the posterior distribution. The short-term forecasts are made from the resulting MCMC samples. We perform the rolling-origin cross-validation procedure to compare the prediction error of different stochastic models. In terms of regions, we used the top 20 countries in terms of confirmed case numbers for a country-level analysis. Observations included that 1) as the models learned more, the predictive performance improved in general for all regions; 2) none of the models proved to be the golden standard across all the regions, while the ARIMA model underperformed all stochastic models proposed in the paper. We designed a graphical interface that allows users to interact with future trends of COVID-19 at different geographic locations in the U.S. based on the real-time COVID-19 data. This web portal is updated daily and used to inform local policy-makers and the general public (https://qiwei.shinyapps.io/PredictCOVID19/).

Data Description

Let $\boldsymbol{C} = (C_1, \ldots, C_T)$ be a sequence of cumulative confirmed case numbers observed at T successive equally spaced points in time (e.g. day) in a specific region, where each entry $C_t \in \mathbb{N}$ for $t = 1, \ldots, T$. Further let C_0 be the initial value and $\dot{\boldsymbol{C}} = (\dot{C}_1, \ldots, \dot{C}_T)$ be the lag one difference of $\boldsymbol{C}$, where $\dot{C}_1 = C_1 - C_0$ and each following entry $\dot{C}_t = C_t - C_{t-1}, t = 2, \ldots, T$, i.e. the difference between two adjacent observations. In the analysis and modeling of a series of infectious disease daily report data, the time-series data could also be the cumulative death numbers, recovery case numbers, or their sums, denoted by $\boldsymbol{D}$ (Death), $\boldsymbol{E}$ (Recovery), and $\boldsymbol{R}$ (Removed), and their corresponding new case numbers, denoted by $\dot{\boldsymbol{D}}$, $\dot{\boldsymbol{E}}$, and $\dot{\boldsymbol{R}}$. Assuming a closed population with size N, the time-series data could also be the number of susceptible people, denoted by $\boldsymbol{S}$, with each entry $S_t = N - C_t$. In reality, only confirmed cases and deaths are reported in most regions. Recovery data are not available or suffer from under-reporting issues even if existing. Thus, our main goal is to make predictions of the future trend of an infectious diseases only based on the daily confirmed cases $\dot{\boldsymbol{C}}$.

Analyses

In this section, we discuss the findings of COVID-19 data analysis. We first implemented each of the growth models listed in Table 1 and the standard SIR model under the proposed Bayesian framework for the top 20 countries with the most confirmed cases as of August 22, 2020. Note that the input data was the sequence of daily confirmed cases $\dot{\boldsymbol{C}}$ only, which is accessible from the Johns Hopkins University Center for Systems Science and Engineering COVID-19 Data Repository (https://github.com/CSSEGISandData/COVID-19/). Several recent COVID-19 studies also based their analyses on this resource [25, 26, 27]. For our MCMC algorithms, we set 100,000 iterations with the first half as burn in and chose weakly informative priors. Both numerical and graphical summaries for posterior inference and short-term forecasting are presented. Our final goal is to compare the predictive performance of all models, taking ARIMA as a benchmark model.

Forecasting of daily confirmed cases in the U.S.

We first present the forecasting of the U.S. daily confirmed cases made by the ARIMA and our Bayesian framework with the choices of a GRC or SIR model. As we can see from Figures 1, the GRC model demonstrates a downwards trend, the SIR model displays an upward trend, while the ARIMA model predicts a flat trajectory of daily predicted cases. A natural feature of epidemiological interest is the estimated final size and date of the epidemic. Growth models comprise of a model parameter K that estimates the final epidemic size. On the other hand, for the SIR model, there is no available parameter that estimates the final size. Hence, the final case count is approximated to be the predictive mean that converges to a certain value from the related MCMC samples. A similar strategy is applied to obtain the predicted mean of the final case counts using the ARIMA model [2]. The estimated cumulative confirmed cases by the end of 2020 are projected to be 13.1, 106.1, and 10.0 (in millions), fitting the GRC, SIR, and ARIMA models, respectively. Under the assumption that the epidemic lasts until the end of 2021, the final epidemic sizes are predicted to be 13.4, 187.3, and 22.0 (in millions) by the three models, respectively. To account for the reasons behind the discrepancy in forecasts and to measure the validity of the results, there is a need for an appropriate strategy to evaluate and compare the predictive performance of the concerned models.

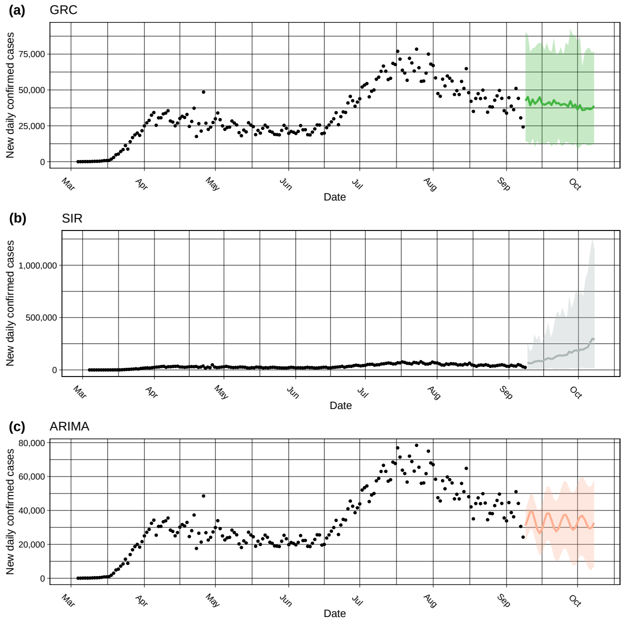

Figure 1. The one-month forecasting of new daily confirmed cases in the U.S. made by the (a) GRC and (b) SIR model under the proposed Bayesian framework, as well as the benchmark (c) ARIMA model. The black circles represent the observed case numbers since the beginning of March, 2020, while the colored circles and ribbons represent the predicted means and 95% prediction intervals, respectively.

Model comparison through rolling-origin cross-validation

Cross-validation (CV) is a resampling procedure used to evaluate regression and classification models on a limited data sample. The procedure randomly splits all data samples into two parts: training and testing sets, where the former is used to fit a model and the latter is used to evaluate the model's prediction performance in terms of a certain error measure. The key assumption of CV is that all data points should be independent and identically distributed (i.i.d.). Unfortunately, time-series data is serially autocorrelated i.e. the observations are dependent on the time they were recorded on. To circumvent this situation, the rolling-origin CV (ROCV) technique was proposed [28]. It splits the data into training and testing sets without hampering the i.i.d. assumption. An adaptation of this method is used here to evaluate the short-term forecasting performance among different top-level choices under the proposed Bayesian framework and ARIMA. Figure 2 shows the ROCV representation for an example of time-series data ($T = 17$). In our analysis, the choice of initial training sample size was set to seven days so as to evaluate how well the models are able to generate forecasts during the initial phase of the pandemic, while the testing sample size was chosen to be three days to meet with our objective of comparing short-term forecasting performance. We define the first day $t = 1$ as the date when the 100-th case was confirmed, so it varied for different countries.

A CV algorithm needs a predictive error metric that could quantify model performance in terms of forecasting accuracy. *Root mean square error* (RMSE) and *mean absolute deviations* (MAD) are candidates of error measures for out-of-bag predictions but are dependent on scale. As a result, large values may influence the errors to be larger. *Mean absolute percentage error* (MAPE) has been a widely used predictive measure due to its interpretability and its independence from scale. Although, the distribution of such percentage errors can be skewed if the

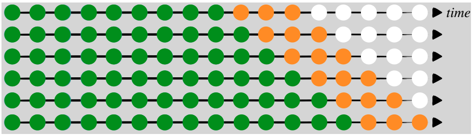

Figure 2. A visual guide to rolling-origin cross-validation (ROCV), where the total sample size $T = 17$, the initial training sample size is 9, and the testing sample size is 3. The green, orange, and white circles are training, testing, and unused samples in one CV iteration.

Algorithm 1 Rolling-origin cross-validation (ROCV)

Store the data starting day 1 to day T
Initialize the number of initial training observations k ($k = 7$)
Set the size of the testing set ω ($\omega = 3$)
while $k + \omega \leq T$ **do**
  Learn the first k observations (green circles) as training data
  Hold out the next $k + 1, \dots, k + \omega$ observations (orange circles) as the testing data
  Discard the remaining $T - (k + \omega)$ observations (white circles)
  Compute an out-of-bag prediction error measure on the testing set (orange circles)

$$\text{sMAPE} = \frac{2}{\omega} \sum_{t=k+1}^{k+\omega} \left| \frac{e_t}{\hat{y}_t + y_t} \right|$$

  $k = k + 1$
end while

data consists of values close to zero. Moreover, there is a possibility of this measure being undefined by having a zero in the denominator. To address these issues, an improved percentage error metric namely, *symmetric mean absolute percentage error* (sMAPE) was proposed [28]. This metric was considered in our analysis as it circumvented the problem of having an undefined measure and provided better symmetry as compared to MAPE. In all, we summarize the evaluation procedure used in this paper as follows.

Figure 3 displays the smoothed sMAPE curves generated by the ROCV across time for the top 20 countries with respect to the highest cumulative confirmed cases as of August 22, 2020. As we can see, all the models performed poorly in the early stage but as more and more data became available to be learned, the predictive performance gradually improved as the sMAPE dropped. It can also be observed that the ARIMA and SIR models were performing a lot worse in general than the growth models in the early phase. This could be attributed to the fact that ARIMA not having the growth specific parameters, unable to detect the early growth. On the other hand, making assumptions of a fixed transmission rate γ and due to the underreported data issue, SIR performed poorly. While, the stochastic growth curves were able to learn the trend of epidemiological data in the initial phase with the help of the growth and scaling parameters. Although, towards the latter half of the epidemic, all the models were performing equally well. Hence, it is hard to conclude that any one particular dominated the entire duration of the epidemic.

Now, the question arises of whether we could pick one model which has the best predictive performance on an average for any particular country. To answer that we construct a Cleveland dot plot, as shown in Figure 4, that allows us to

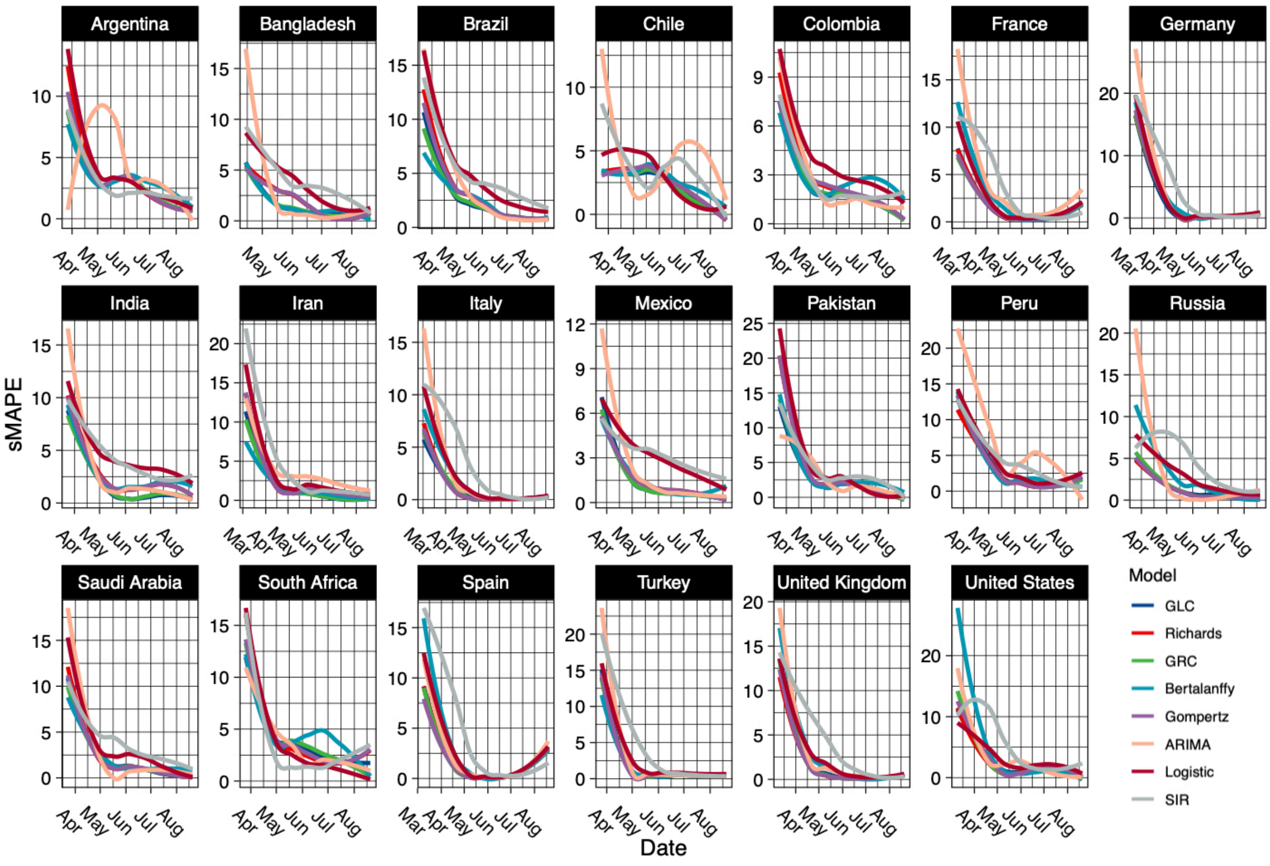

Figure 3. The smoothed sMAPE curves generated by the rolling-origin cross-validation (ROCV) over time for the top 20 countries with the most confirmed cases as of August 22, 2020.

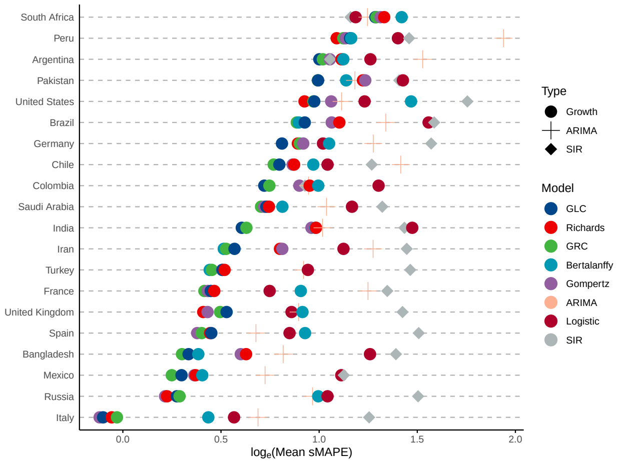

Figure 4. The Cleveland dot plot of the averaged sMAPE generated by the rolling-origin cross-validation (ROCV) for the top 20 countries with the most confirmed cases as of August 22, 2020.

rank the model performance averaged over the entire duration, per country. Furthermore, the countries are arranged in descending order of predictive performance from the bottom to the top.

Discussion

It is observed that all the models had the best and the worst predictive performances for Italy and South Africa respectively. The Richards model had the minimum averaged sMAPE for forecasting cumulative case counts in the U.S. While, the GRC model had the lowest averaged sMAPE across seven countries followed by the GLC model with four. The SIR model was the best performer for South Africa. The Richards, Bertalanffy, and Gompertz models also had their fair share of predictive dominance in the remaining countries. On the other hand, the ARIMA model was a below-average performer across all countries.

In general, the GRC and GLC models were consistent performers throughout all countries due to their ability to detect sub-exponential growth rates at an early stage of an epidemic. In most cases, the GRC and Richards models were the best performers in countries that did not have symmetric 'S'-shaped growth patterns and displayed randomness as well as multiple peaks. This is due to the inclusion of the scale parameter α that could account for any asymmetry in the data. Countries including the U.S., Peru, Saudi Arabia, Iran, Turkey, and France display multiple peaks in the daily confirmed case counts. As a result, the Richards model performs the best in the U.S., United Kingdom (U.K.), and Peru, while the GRC model dominates in the rest of the countries having multiple peaks. Moreover, a random structure was observed in countries like Brazil, Chile, Bangladesh, and Mexico. GRC being the most complex model out of all the other growth models performed the best in these countries. On the other hand, the GLC model usually performed better in countries that had a single peak and attributed an approximate 'S'-shaped curvature. The GLC model was able to generalize better than the GRC model when the data was well structured and had less randomness. Countries including Argentina, Pakistan, Germany, Colombia, and India attributed a single peak without much randomness. As a result, the GLC

model was a better performer in these countries. Whereas, in the case of South Africa, the usual growth models performed the worst due to a staggering growth rate in the initial and the middle phase of the epidemic. The SIR model performed the best out of the worst while the logistic model performed well due to its simplicity. On the other hand, the Gompertz model was the best performer in Russia, Spain, and Italy as it generalizes better than the other models.

Conclusion

In this paper, we developed a number of stochastic variants of growth and compartmental models under a unified Bayesian framework. A theoretical comparison of growth models has been discussed in greater detail in the literature [5, 6, 7, 29, 30, 31, 32, 33]. However, to our best knowledge, no work systematically compares their performances between all pairs as well as against a compartmental model such as the SIR model and a time-series forecasting model such as the ARIMA. Based on our analysis, we conclude that the proposed Bayesian framework not only allows room for interpretation but also offers an exemplary predictive performance when it comes to COVID-19 daily report data. Moreover, ARIMA being a pure learning algorithm is not able to match with the forecasting accuracy of stochastic models, let alone the model parameters of ARIMA do not provide any information on epidemiological interests.

For future work, we aim to develop an ensemble model, which can aggregate the prediction of each base model and results in once final prediction for the unseen data. Note that a group of researchers have recently introduced a GGM-GLM ensemble model [32] and compared forecasting performances of that with the individual models for the Ebola Forecasting Challenge [34]. It was reported that the ensemble model outperformed the others under some circumstances. We also plan to perform long-term forecasting evaluation using some epidemic features described in [24]. A sub-epidemic wave model that could detect multiple peaks in the data has been recently developed [35], which has the potential to improve forecasting performance. Thus, developing stochastic growth models via the addition of a change-point detection mechanism to account for multiple peaks is worth investigating. In this regard, we have demonstrated that an approach that combines a change-point detection model and a stochastic SIR model could significantly improve the short-term forecasting of the new daily confirmed cases [36].

Potential Implications

The proposed Bayesian epidemiological models in a unified framework lay the foundation of an integrative approach to model and predict epidemiological data with tremendous accuracy and interpretability. Growth and compartmental models obtained as solutions to ordinary differential equations (ODEs) are implemented to model epidemiological data under a deterministic setting as they provide a natural framework representative of such data types. However, the estimated model parameters crucial for providing insights into the nature of the epidemic are unreliable under the deterministic setting due to identifiability issues. The stochastic models mimic the structure of epidemiological models and incorporate parameter specific priors and measurement error to solve these issues. Researchers can follow a similar setup to predict cases and deaths caused by an epidemic at any geographical level given the availability of data. Furthermore, the stochastic SIR model can be augmented by incorporating mobility, hospitalization, and recovery data resulting in better forecasts. This work also promotes an algorithmic strategy to measure forecasting performances of time series models in general.

On a much broader scale, this work encourages researchers to exploring probabilistic approaches to model epidemiological data as well as developing computationally efficient algorithms that further meet time and cost constraints.

Methods

In this section, we present a bi-level Bayesian framework for predicting new daily confirmed cases during a pandemic in a closed society. The bottom level directly models the observed counts while accounting for measurement errors. Two alternatives for the top level are then introduced, both of which characterizes the epidemic dynamics through growth curve or compartmental trajectories, respectively. Before introducing the main components, we summarize the possibly observable data as follows.

Bottom-level: Time-series count generating process

We consider the new case number observed at time t, i.e. $\dot{C}_t$, are sampled from a negative binomial (NB) model,

$$\dot{C}_t \sim \text{NB}(g(C_{t-1}, \Theta), \phi), \quad t = 2, \ldots, T$$

as it automatically accounts for measurement errors and uncertainties associated with the counts. Here, we use $\text{NB}(\mu, \phi), \mu, \phi > 0$ to denote a NB distribution with expectation μ and dispersion $1/\phi$. We assume this stochastic process is a Markov process, where the present state (at time t) depends only upon its previous state (at time $t-1$). Therefore, the NB mean is a function, denoted by $g(\cdot)$, of the case number observed at time $t-1$, characterized by a set of interpretable/uninterpretable model parameters Θ. With this parameterization, the NB variance is $\mu + \mu^2/\phi$, indicating that ϕ controls the variance of measurement error. A small value leads to a large variance to mean ratio, while a large value approaching infinity reduces the NB model to a Poisson model with the same mean and variance. The probability mass function of a NB random variable Y is $\frac{\Gamma(Y+\phi)}{Y!\Gamma(\phi)}\left(\frac{\phi}{\lambda+\phi}\right)^{\phi}\left(\frac{\lambda}{\lambda+\phi}\right)^{Y}$. Thus, we can write the full data likelihood as

$$f(\dot{\boldsymbol{C}}|\Theta, \phi) = \prod_{t=2}^{T} \frac{\Gamma(\dot{C}_t + \phi)}{\dot{C}_t!\Gamma(\phi)} \left(\frac{\phi}{g(C_{t-1}, \Theta) + \phi}\right)^{\phi} \left(\frac{g(C_{t-1}, \Theta)}{g(C_{t-1}, \Theta) + \phi}\right)^{\dot{C}_t}. \quad (1)$$

For the prior distribution of the dispersion parameter ϕ, we choose a gamma distribution, $\phi \sim \text{Ga}(a_\phi, b_\phi)$. We recommend small values, such as $a_\phi = b_\phi = 0.001$, for a non-informative setting [37]. Note that the proposed framework can be also viewed as a stochastic discrete-time state-space model with a negative binomial error structure. The proposed Bayesian models, on average, mimics the epidemic dynamics and is more flexible than those deterministic epidemiological models, as it accounts for measurement error and has the potential to incorporate existing information into the prior structure of Θ.

Top-level I: Growth model

We first discuss the choices of $g(\cdot)$ when implementing growth models. The development of a variety of growth curves originates from population dynamics [38] and growth of biological systems [39, 40, 41, 42] modeling. A number of growth

curves have been adapted in epidemiology for trend characterization and forecasting of an epidemic, such as the severe acute respiratory syndrome (SARS) [43, 44], dengue fever [45, 46], pandemic influenza A (H1N1) [47], Ebola virus disease (EVD) [29, 30], Zika fever [31], and COVID-19 [3, 6, 7, 48].

The underlying assumption is that the rate of growth of a population, organism, or infectious individuals eventually declines with size. The logistic curve (also known as sigmoid curve) is the simplest growth curve of continuous time $u \in \mathbb{R}$. It is a non-negative symmetric 'S'-shaped curve with equation $y(u) = \frac{K}{1+\exp(-\lambda(u-u_0))}$, where u_0 is the midpoint, K is the maximum value, and λ reflects the steepness of the curve. It is clear to see that $y(u)$ approaches K when $u \to \infty$, while it converges to zero when $u \to -\infty$. In fact, the continuous curve $y(u)$ is the solution of a first-order non-linear ODE,

$$\frac{dy(u)}{du} = \lambda y(u)\left(1 - \frac{y(u)}{K}\right)$$

with condition $y(u_0) = K/2$, where $dy(u)/du$ can be interpreted as time-variant growth rate of the curve y. The above ODE reveals: 1) a non-negative growth rate, $dy(u)/du > 0$ as $y(u) \in [0, K]$; 2) an approximately exponential growth at the initial stage, $y(u) \approx \exp(\lambda u)$ as $dy(u)/du \approx \lambda y(u)$ when $y(u) \to 0$; 3) no growth at the final stage, $y(u)\ dy(u)/du = 0$ when $y(u) \to K$; 4) a maximum growth rate of $\lambda K/4$ occurred when $y(u) = K/2$, indicated by $d^2y(u)/du^2 = \lambda dy(u)/du\,(1 - 2y(u)/K)$. Based on those curve characteristics, we can use the growth curve to characterize the trend of cumulative confirmed cases $\mathbf{C}$.

In this paper, we mainly consider a family of growth curves that are derived from the generalized Richards curve (GRC), which is the solution to the following ODE,

$$\frac{dy(u)}{du} = \lambda y(u)^p \left[1 - \left(\frac{y(u)}{K}\right)^{\alpha}\right]. \tag{2}$$

For those model-specific parameters in the context of epidemiology, K is the final epidemic size and should be an integer in the range of $(0, N]$, where N is the total population, $\lambda \in \mathbb{R}^+$ is the infectious rate at early epidemic stage, $p \in (0, 1)$ is known as scaling of growth, and $\alpha \in \mathbb{R}^+$ controls the curve symmetry. As our observed infectious disease data are usually counts collected at successive equally spaced discrete time points, we formulate the NB mean function $g(\cdot)$ based on the discrete version of (2),

$$g(C_{t-1}, \Theta = \{K, \lambda, p, \alpha\}) = \lambda C_{t-1}^p \left[1 - \left(\frac{C_{t-1}}{K}\right)^{\alpha}\right]. \tag{3}$$

Table 1 provides a list of $g(\cdot)$'s for growth curves with their characteristics. All the listed growth curves have been utilized and discussed in previous epidemiological studies. We include all of those choices in our framework excluding the last one, which is based on the generalized growth curve (GGC), because it lacks the final epidemic size K specification.

Without any existing information, we assume that K is from a discrete uniform distribution in its range and γ is from a gamma or a beta distribution, depending on the choice of growth curves. For instance, for both logistic and Gompertz curves, we assume $\gamma \sim \text{Beta}(a_\gamma, b_\gamma)$, a natural modeling choice for parameter value restricted to the $(0, 1)$ interval, and suggest to choose $a_\gamma = b_\gamma = 1$ for a uniform setting; otherwise, we place a gamma prior, i.e. $\gamma \sim \text{Ga}(a_\gamma = 0.001, b_\gamma = 0.001)$. For the choice of GRC and generalized logistic curve (GLC), the prior of p is chose to be $\text{Beta}(a_p = 1, b_p = 1)$. Lastly, we set $\alpha \sim \text{Ga}(a_\gamma = 0.001, b_\gamma = 0.001)$ for fitting a GRC or Richards curve.

Top-level II: Compartmental model

The susceptible-infected-removed (SIR) model was developed to simplify the mathematical modeling of human-to-human infectious diseases by Kermack and McKendrick [50]. It is a fundamental compartmental model in epidemiology. At any given time u, each individual in a closed population with size N is assigned to three distinctive compartments with labels: susceptible (S), infectious (I), or removed (R, being either recovery or dead). The standard SIR model describes the flow of people from S to I and then from I to R by the following set of nonlinear ODEs:

$$\begin{cases} \frac{dS(u)}{du} = -\beta S(u)\frac{I(u)}{N} \\ \frac{dI(u)}{du} = \beta S(u)\frac{I(u)}{N} - \gamma I(u) \\ \frac{dR(u)}{du} = \gamma I(u) \end{cases},$$

where $S(u)$, $I(u)$, and $R(u)$ are the population numbers of susceptible, infectious, and removed compartments measured in time u, subjecting to $S(u) + I(u) + R(u) = N, \forall u$. Another nature constraint is $dS(u)/du + dI(u)/du + dR(u)/du = 0$. Here, $\beta \in \mathbb{R}^+$ is the disease transmission rate, $\gamma \in \mathbb{R}^+$ is the removal rate, and their ratio $\mathcal{R}_0 = \beta/\gamma$ is defined as the *basic reproductive number*. The rationale behind the first equation is that the number of new infections during an infinitesimal amount of time, $-dS(u)/du$, is equal to the number of susceptible people, $S(u)$, times the product of the contact rate, $I(t)/N$, and the disease transmission rate β. The third equation reflects that the infectious individuals leave the infectious population per unit time, $dI(u)/du$, as a rate of $\gamma I(u)$. The second equation follows immediately from the first and third ones as a result of $dS(u)/du + dI(u)/du + dR(u)/du = 0$. Assuming that only a small fraction of the population is infected or removed in the onset phase of an epidemic, we have $S(u)/N \approx 1$ and thus the second equation reduces to $dI(u)/du = (\beta - \gamma)I(u)$, revealing that the infectious population is growing if and only if $\beta > \gamma$. As the expected lifetime of an infected case is given by γ^{-1}, the ratio $\mathcal{R}_0 = \beta/\gamma$ is the average number of new infectious cases directly produced by an infected case in a completely susceptible population. The so called basic reproductive number is a good indicator of the transmissibility of an infectious disease.

In this paper, we only consider the standard SIR model, although it is still feasible to design $g(\cdot)$'s from its variations (see a comprehensive summary [51]), such as the susceptible-infectious (SIS) model, the susceptible-infectious-recovered-deceases (SIRD) model, the susceptible-exposed-infectious-removed (SEIR) model, the susceptible-exposed-infectious-susceptible (SEIS) model, and their versions with the maternally-derived immunity compartment [52], as well as the recently developed extended-SIR (eSIR) model [14]. For modeling discrete time-series data, we use the discrete-time version of the standard SIR model,

$$\begin{cases} \dot{S}_t = -\beta S_{t-1}\frac{I_{t-1}}{N} \\ \dot{I}_t = \beta S_{t-1}\frac{I_{t-1}}{N} - \gamma I_{t-1} \\ \dot{R}_t = \gamma I_{t-1} \end{cases}. \tag{4}$$

The model has three trajectories, one for each compartment. The compositional nature of the three trajectories implies that we only need two of the three sequence data, e.g. $S_t = N - C_t$ and R_t for $t = 1, \ldots, n$. However, recovery data only exist in few regions, and suffer from under-reporting issue even if existing, which makes both model inference and predictions infeasible. Alternatively, we consider both of the removed and actively infectious cases as missing data and mimic their relationship in spirit to some compartmental models in epidemiology. Specifically, we assume the number of new removed cases at time t,

Table 1. List of $g(\cdot)$'s functions based on growth curves

Models	$g(C_{t-1}, \Theta)$	Parameters Θ	Curve $y(u)$	$y(u)$ at the turning point	Examples
GRC	$\lambda C_{t-1}^{p}\left[1-\left(\frac{C_{t-1}}{K}\right)^{\alpha}\right]$	$K \in \mathbb{N}$,<br>$\lambda \in \mathbb{R}^{+}$,<br>$p \in (0,1)$,<br>$\alpha \in \mathbb{R}^{+}$	N/A	$\left(\frac{p}{p+\alpha}\right)^{1/\alpha} K$	[7, 29, 31]
Richards	$\lambda C_{t-1}\left[1-\left(\frac{C_{t-1}}{K}\right)^{\alpha}\right]$	$K \in \mathbb{N}$,<br>$\lambda \in \mathbb{R}^{+}$,<br>$\alpha \in \mathbb{R}+$	$K\left(1+A\exp(-\lambda\alpha u)\right)^{-1/\alpha}$,<br>where $A = -1+\left(\frac{K}{y(0)}\right)^{\alpha}$	$\left(\frac{1}{1+\alpha}\right)^{1/\alpha} K$	[43, 44, 45, 46, 47]
GLC	$\lambda C_{t-1}^{p}\left(1-\frac{C_{t-1}}{K}\right)$	$K \in \mathbb{N}$,<br>$\lambda \in \mathbb{R}^{+}$,<br>$p \in (0,1)$	N/A	$\frac{p}{p+1}K$	[7, 35, 32, 33]
Logistic	$\lambda C_{t-1}\left(1-\frac{C_{t-1}}{K}\right)$	$K \in \mathbb{N}$,<br>$\lambda \in (0,1)$	$K\left(1+A\exp(-\lambda u)\right)^{-1}$,<br>where $A = -1+\frac{K}{y(0)}$	$\frac{1}{2}K$	[3, 6, 7, 29, 30]
Bertalanffy	$\lambda C_{t-1}^{\frac{2}{3}}\left[1-\left(\frac{C_{t-1}}{K}\right)^{\frac{1}{3}}\right]$	$K \in \mathbb{N}$,<br>$\lambda \in \mathbb{R}^{+}$	$K\left(1+A\exp(-\frac{1}{3}\gamma K^{-1/3}u)\right)^{3}$,<br>where $A = 1-\left(\frac{y(0)}{K}\right)^{1/3}$	$\frac{8}{27}K$	[6]
Gompertz	$\lambda C_{t-1}\log\frac{K}{C_{t-1}}$	$K \in \mathbb{N}$,<br>$\lambda \in (0,1)$	$K\exp(A\exp(-\lambda u))$,<br>where $A = \log\frac{y(0)}{K}$	$\frac{1}{e}K$	[6]
GGC	λC_{t-1}^{p}	$\lambda \in \mathbb{R}^{+}$,<br>$p \in (0,1)$	$(A+\lambda u(1-p))^{1/(1-p)}$<br>where $A = y(0)^{1-p}$	N/A	[7, 31, 32, 33, 49]

Abbreviations: GRC is generalized Richards curve; GLC is generalized logistic curve; GGC is generalized growth curve.

i.e. $\dot{R}_t$, is sampled from a Poisson distribution with mean γI_{t-1}, that is, $\dot{R}_t \sim \text{Poi}(\gamma I_{t-1}) = \text{Poi}(\gamma(N - C_{t-1} - R_{t-1}))$, where γ should be specified. Such a strategy but with different error structure was also considered in some other compartmental models in epidemiology [16, 53, 54]. We can estimate the value of γ from publicly available high-quality data where confirmed, deaths, and recovery cases are all well-documented, or from prior epidemic studies due to the same under-reporting issue in actual data. In this paper, we choose the removal rate $\gamma = 0.1$ as suggested by Pedersen and Meneghini [55] and Weitz et al. [56]. Based on this simplification, we rewrite the first equation in (4) as,

$$(N - C_t) - (N - C_{t-1}) \quad = \quad -\beta(N - C_{t-1})\frac{N - C_{t-1} - R_{t-1}}{N},$$

resulting in

$$\dot{C}_t \quad = \quad \beta(N - C_{t-1})\frac{N - C_{t-1} - R_{t-1}}{N}.$$

Thus, we formulate the NB mean function $g(\cdot)$ for the standard SIR model as,

$$g(C_{t-1}, \Theta = \{\beta\}|\boldsymbol{R}) \quad = \quad \beta(N - C_{t-1})\frac{N - C_{t-1} - R_{t-1}}{N}, \qquad (5)$$

where $\boldsymbol{R}$ can be sequentially inferred from $\boldsymbol{C}$.

Without any existing information, in our Bayesian framework we assume β from a gamma distribution with hyperparameters that makes both the mean and variance of the transformed variable $\mathcal{R}_0 = \beta/\gamma$ equal to 1, that is, $\beta \sim \text{Ga}(1, 1/\gamma)$.

Model Fitting

In this section, we briefly describe the MCMC algorithm for posterior inference and forecasting. Our Bayesian inferential strategy allows us to simultaneously infer all model-specific parameters and quantify their uncertainties.

MCMC algorithms

We first describe how to update the dispersion parameter ϕ in the proposed Bayesian framework, as it does not depend on the choice of models. At each MCMC iteration, we perform the following step:

Update of dispersion parameter ϕ: We update ϕ by using a random walk Metropolis-Hastings (RWMH) algorithm. We first propose a new ϕ^*, of which logarithmic value is generated from $\text{N}\left(\log\phi, \tau_\phi^2\right)$ and then accept the proposed value ϕ^* with probability $\min(1, m_{\text{MH}})$, where the Hastings ratio is

$$m_{\text{MH}} \quad = \quad \frac{f(\dot{\boldsymbol{C}}|\phi^*, \Theta)}{f(\dot{\boldsymbol{C}}|\phi, \Theta)}\frac{\pi(\phi^*)}{\pi(\phi)}\frac{J(\phi \leftarrow \phi^*)}{J(\phi^* \leftarrow \phi)}.$$

Here we use $J(\cdot \leftarrow \cdot)$ to denote the proposal probability distribution for the selected move. Note that the last term, which is the proposal density ratio, cancels out for this RWMH update.

Top-level as a growth model

We only present the updates of each parameters in the GRC model, as all other derivative models are its special cases. Our primary interest lies in the estimation of the final pandemic size K and the infectious rate at early epidemic stage λ.

Update of final epidemic size parameter K: We update K by using a RWMH algorithm. We first propose a new K^*, of which logarithmic value is generated from a truncated Poisson distri-

bution Poi $(\log K)$ within $[\log C_T, \log N]$ and then accept the proposed value K^* with probability $\min(1, m_{MH})$, where the Hastings ratio is

$$m_{MH} = \frac{f(\dot{C}|\phi, K^*, \lambda, p, \alpha)}{f(\dot{C}|\phi, K, \lambda, p, \alpha)} \frac{\pi(K^*)}{\pi(K)} \frac{J(K \leftarrow K^*)}{J(K^* \leftarrow K)}.$$

Note that with a discrete uniform prior on K, the last two terms cancel out for this RWMH update.

Update of infectious rate parameter λ: We update λ by using a RWMH algorithm. We first propose a new λ^*, of which logarithmic value is generated from $N\left(\log\lambda, \tau_\lambda^2\right)$ and then accept the proposed value λ^* with probability $\min(1, m_{MH})$, where the Hastings ratio is

$$m_{MH} = \frac{f(\dot{C}|\phi, K, \lambda^*, p, \alpha)}{f(\dot{C}|\phi, K, \lambda, p, \alpha)} \frac{\pi(\lambda^*)}{\pi(\lambda)} \frac{J(\lambda \leftarrow \lambda^*)}{J(\lambda^* \leftarrow \lambda)}.$$

Note that the last term, which is the proposal density ratio, cancels out for this RWMH update.

Update of growth scaling parameter p: We update p by using a RWMH algorithm. We first propose a new p^*, of which logarithmic value is generated from a truncated normal distribution $N\left(\log p, \tau_p^2\right)$ within $[-\infty, 0]$ and then accept the proposed value p^* with probability $\min(1, m_{MH})$, where the Hastings ratio is

$$m_{MH} = \frac{f(\dot{C}|\phi, K, \lambda, p^*, \alpha)}{f(\dot{C}|\phi, K, \lambda, p, \alpha)} \frac{\pi(p^*)}{\pi(p)} \frac{J(p \leftarrow p^*)}{J(p^* \leftarrow p)}.$$

Note that with a uniform prior on p over its range $[0, 1]$, the last two terms cancel out for this RWMH update.

Update of symmetry parameter α: We update α by using a RWMH algorithm. We first propose a new α^*, of which logarithmic value is generated from $N\left(\log\alpha, \tau_\alpha^2\right)$ and then accept the proposed value α^* with probability $\min(1, m_{MH})$, where the Hastings ratio is

$$m_{MH} = \frac{f(\dot{C}|\phi, K, \lambda, p, \alpha^*)}{f(\dot{C}|\phi, K, \lambda, p, \alpha)} \frac{\pi(\alpha^*)}{\pi(\alpha)} \frac{J(\alpha \leftarrow \alpha^*)}{J(\alpha^* \leftarrow \alpha)}.$$

Note that the last term, which is the proposal density ratio, cancels out for this RWMH update.

Top-level as a compartmental model

Our primary interest lies in the estimation of the reproductive number $\mathcal{R}_0 = \beta/\gamma$, which reflects the transmissibility of the disease. With our assumption that γ is given, we propose the following updates in each MCMC iterations.

Generate R **based on** C**:** We assume $I_1 = C_1$, i.e. all the confirmed cases are capable to pass the disease to all susceptible individuals in a closed population at the very beginning. In other words, $R_1 = 0$. Then we sample $R_2 \sim \text{Poi}(\gamma I_1)$, where γ is a pre-specified tuning parameter. Due to the compositional nature, we can compute $I_2 = I_1 + \dot{C}_2 - R_2$, where $\dot{C}_2 = C_2 - C_1$ is the new confirmed cases and R_1 is the total removed cases from the actively infectious population at time 2. Next, we repeat this process of sampling $R_t \sim \text{Poi}(\gamma I_{t-1})$ and computing $I_t = I_{t-1} + \dot{C}_t - R_t$, to generate the sequence $\boldsymbol{R}$.

Update of reproduction number parameter β: We update β by using a RWMH algorithm. We first propose a new β^*, of which logarithmic value is generated from a truncated normal distribution $N\left(\log\beta, \tau_\beta^2\right)$ and then accept the proposed value β^* with probability $\min(1, m_{MH})$, where the Hastings ratio is

$$m_{MH} = \frac{f(\dot{C}|\beta^*, \boldsymbol{R})}{f(\dot{C}|\beta, \boldsymbol{R})} \frac{\pi(\beta^*)}{\pi(\beta)} \frac{J(\beta \leftarrow \beta^*)}{J(\beta^* \leftarrow \beta)}.$$

Note that the last term, which is the proposal density ratio, cancels out for this RWMH update.

Posterior inference

We obtain posterior inference by post-processing the MCMC samples after burn-in. Suppose that a sequence of MCMC samples on $\theta, \theta \in \{\phi, K, \lambda, p, \alpha, \beta\}$,

$$\theta^{(1)}, \ldots, \theta^{(U)}$$

have been collected, where $u, u = 1, \ldots, U$ indexes the iteration after burn-in. An approximate Bayesian estimator of each parameter can be simply obtained by averaging over the samples, $\hat{\theta} = \sum_{u=1}^{U} \theta^{(u)}/U$. In additional to that, a quantile estimation or credible interval for each parameter of interest can be obtained from this sequence as well.

Forecasting

Based on the sequences of MCMC samples on K, λ, p, and α in the growth model or β in the compartmental model, we can predict the cumulative or new confirmed cases at any future time T_f by Monte Carlo simulation. Particularly, from time $T+1$ to T_f, we sequentially generate

$$\dot{C}_t^{(u)} \sim \text{NB}(g(C_{t-1}, \Theta^{(u)}), \phi^{(u)}), \quad t = T+1, \ldots, T_f. \quad (6)$$

Then, both short and long-term forecast can be made by summarizing the $(T_f - T)$-by-U matrix of MCMC samples. For instance, the predictive number of cumulative and new confirmed cases at time $T+1$, in average, are $\sum_{u=1}^{U} C_{T+1}^{(u)}/U$ and $\sum_{u=1}^{U} \dot{C}_{T+1}^{(u)}/U$, respectively.

Software

This paper introduces a user-friendly interactive web application (`https://qiwei.shinyapps.io/PredictCOVID19/`) integrated with R `Shiny` package. `Shiny` is a web platform that allows users to interact with real-time data and use a myriad of visualization tools to analyze it. The web application has been developed to help the general public assess both short and long-term forecasts of COVID-19 across the U.S. at both state and metropolitan-level. The numbers of cumulative or new daily confirmed cases as well as deaths are projected by different growth models and the SIR model under the proposed Bayesian framework. Alongside the numerical summaries, users can view and interpret the trends that cover the same information. To validate the short-term forecasting, numerical and graphical summaries of MAE and MAPE of the predictions are provided for the more advanced users. Moving on to the long-term forecasting, the models estimate the peak number of cases and deaths as well as their respective dates. Moreover, a predictive estimate for the final size and date is also offered. Finally, for the users that are keen to visualize the currently observed cases at a geographical level, the website offers county-level spatial maps.

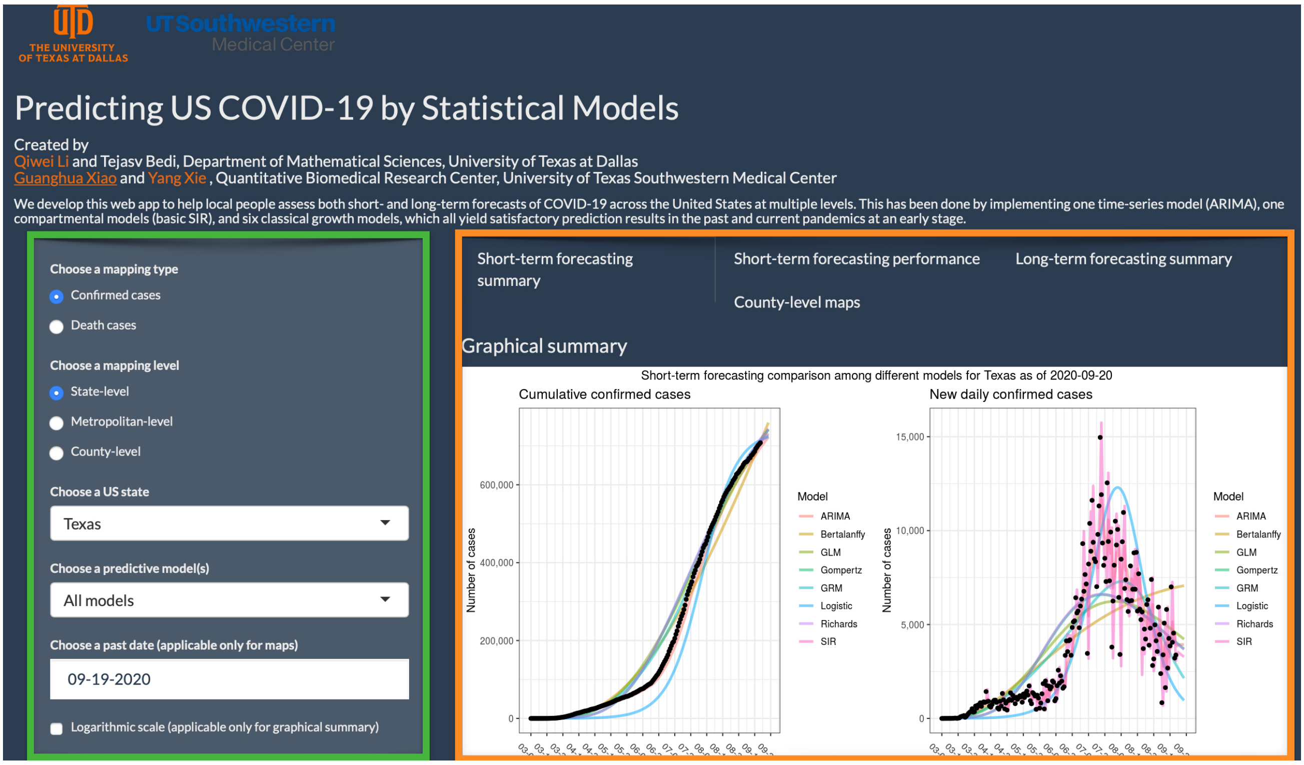

Figure 5. The web interface of the COVID-19 trend analysis page. The green box highlights the input panel that allows users to choose different mapping types and levels for a region. The orange box highlights the visualizations for short-term forecasting as per the instructions of users. Other tabs offer different graphs for summarizing the model performance, long-term forecasting, and county-level spatial maps.

Availability of source code and requirements

- Project name: BayesEpiModels
- Project home page: `https://github.com/liqiwei2000/BayesEpiModels`
- Operating system(s): Windows and Linux
- Programming language: `R` (version 3.6.0.)
- Other requirements: Not applicable.
- License: GNU General Public License v3.0

Availability of supporting data and materials

The related `R/C++` codes for model preparation and execution are available on GitHub at `https://github.com/liqiwei2000/BayesEpiModels`. The `R Shiny` web application is available for users at `https://qiwei.shinyapps.io/PredictCOVID19/`. The COVID-19 data repository is operated by the Johns Hopkins University Center for Systems Science and Engineering (JHU CSSE) and is freely available on GitHub at `https://github.com/CSSEGISandData/COVID-19/`.

List of abbreviations

AI: Artificial Intelligence, ARIMA: Autoregressive integrated moving average, COVID-19: Coronavirus disease - 2019, CV: Cross-validation, eSIR: extended susceptible-infectious-removed, GGC: Generalized growth curve, GGM-GLM: Generalized growth model - Generalized logistic model, GLC: Generalized logistic curve, GRC: Generalized Richards curve, i.i.d.: independent and identically distributed, JHUCSSE: Johns Hopkins University Center for Systems Science and Engineering, MAD: Mean absolute deviations, MAPE: Mean absolute percentage error, MCMC: Markov chain Monte Carlo, NB: Negative Binomial, ODE: Ordinary differential equation, RMSE: Root mean square error, ROCV: Rolling-origin cross-validation, RWMH: Random walk Metropolis-Hastings, SARS-Cov-2: Severe acute respiratory syndrome coronavirus 2, SEIR: susceptible-exposed-infectious-removed, SEIS: susceptible-exposed-infectious-susceptible, SIR: susceptible-infectious-removed, SIRD: susceptible-infectious-recovered-deceases, SIS: susceptible-infectious-susceptible, sMAPE: symmetric Mean Absolute Percentage Error, U.K.: United Kingdom, U.S.: United States.

Ethical Approval

Not applicable.

Consent for publication

Not applicable.

Competing Interests

The authors declare that they have no competing interests.

Funding

This work was partially supported by the National Institutes of Health [R35GM136375, P30CA142543, 5R01CA152301, P50CA70907, 1R01GM115473]; and the Cancer Prevention and Research Institute of Texas [RP190107, RP180805].

Author's Contributions

QL has developed the Bayesian framework, designed the MCMC algorithms, and constructed the `R Shiny` web application. QL

and TB have contributed to the review of different methods and collaborated in the real data analysis. QL, CL, GX, and YX have conceived the study and supervised the web application development and the statistical analyses. All authors have contributed to the writing of the manuscript. All authors have read and approved the final manuscript.

Acknowledgements

The authors would like to thank Drs. Swati Biswas, Min Chen, Pankaj Choudhary, and Vladimir Dragovic at the University of Texas at Dallas for their support and valuable suggestions on this work.

References

1. Elmousalami HH, Hassanien AE. Day level forecasting for Coronavirus Disease (COVID-19) spread: analysis, modeling and recommendations. arXiv preprint arXiv:200307778 2020;.
2. Perone G. An ARIMA model to forecast the spread and the final size of COVID-2019 epidemic in Italy. medRxiv 2020;.
3. Batista M. Estimation of the final size of the COVID-19 epidemic. medRxiv doi 2020;10:16–20023606.
4. COVID I, Murray CJ, et al. Forecasting COVID-19 impact on hospital bed-days, ICU-days, ventilator-days and deaths by US state in the next 4 months. medRxiv 2020;.
5. Roosa K, Lee Y, Luo R, Kirpich A, Rothenberg R, Hyman J, et al. Real-time forecasts of the COVID-19 epidemic in China from February 5th to February 24th, 2020. Infectious Disease Modelling 2020;5:256–263.
6. Jia L, Li K, Jiang Y, Guo X, et al. Prediction and analysis of Coronavirus Disease 2019. arXiv preprint arXiv:200305447 2020;.
7. Wu K, Darcet D, Wang Q, Sornette D. Generalized logistic growth modeling of the COVID-19 outbreak in 29 provinces in China and in the rest of the world. arXiv preprint arXiv:200305681 2020;.
8. Fanelli D, Piazza F. Analysis and forecast of COVID-19 spreading in China, Italy and France. Chaos, Solitons & Fractals 2020;134:109761.
9. Kucharski AJ, Russell TW, Diamond C, Liu Y, Edmunds J, Funk S, et al. Early dynamics of transmission and control of COVID-19: A mathematical modelling study. The Lancet Infectious Diseases 2020;.
10. Li R, Pei S, Chen B, Song Y, Zhang T, Yang W, et al. Substantial undocumented infection facilitates the rapid dissemination of novel coronavirus (SARS-CoV-2). Science 2020;368(6490):489–493.
11. Liu Z, Magal P, Seydi O, Webb G. Predicting the cumulative number of cases for the COVID-19 epidemic in China from early data. arXiv preprint arXiv:200212298 2020;.
12. Pan A, Liu L, Wang C, Guo H, Hao X, Wang Q, et al. Association of public health interventions with the epidemiology of the COVID-19 outbreak in Wuhan, China. Journal of American Medical Association 2020;323(19):1915–1923.
13. Pei S, Shaman J. Initial Simulation of SARS-CoV2 Spread and Intervention Effects in the Continental US. medRxiv 2020;.
14. Song PX, Wang L, Zhou Y, He J, Zhu B, Wang F, et al. An epidemiological forecast model and software assessing interventions on COVID-19 epidemic in China. medRxiv 2020;.
15. Sun H, Qiu Y, Yan H, Huang Y, Zhu Y, Chen SX. Tracking and predicting COVID-19 epidemic in China Mainland. medRxiv 2020;.
16. Wang L, Wang G, Gao L, Li X, Yu S, Kim M, et al. Spatiotemporal dynamics, nowcasting and forecasting of COVID-19 in the United States. arXiv preprint arXiv:200414103 2020;.
17. Yamana T, Pei S, Shaman J. Projection of COVID-19 Cases and Deaths in the US as Individual States Re-open May 4, 2020. medRxiv 2020;.
18. Yang Z, Zeng Z, Wang K, Wong SS, Liang W, Zanin M, et al. Modified SEIR and AI prediction of the epidemics trend of COVID-19 in China under public health interventions. Journal of Thoracic Disease 2020;12(3):165.
19. Gomez J, Prieto J, Leon E, Rodriguez A. INFEKTA: A General Agent-based Model for Transmission of Infectious Diseases: Studying the COVID-19 Propagation in Bogotá-Colombia. medRxiv 2020;.
20. Bullock J, Pham KH, Lam CSN, Luengo-Oroz M, et al. Mapping the landscape of artificial intelligence applications against COVID-19. arXiv preprint arXiv:200311336 2020;.
21. Hu Z, Ge Q, Jin L, Xiong M. Artificial intelligence forecasting of COVID-19 in China. arXiv preprint arXiv:200207112 2020;.
22. Distante C, Pereira IG, Goncalves LMG, Piscitelli P, Miani A. Forecasting COVID-19 Outbreak Progression in Italian Regions: A model based on neural network training from Chinese data. medRxiv 2020;.
23. Vaishya R, Javaid M, Khan IH, Haleem A. Artificial Intelligence (AI) applications for COVID-19 pandemic. Diabetes & Metabolic Syndrome: Clinical Research & Reviews 2020;.
24. Tabataba FS, Chakraborty P, Ramakrishnan N, Venkatramanan S, Chen J, Lewis B, et al. A framework for evaluating epidemic forecasts. BMC Infectious Diseases 2017;17(1):345.
25. Dong E, Du H, Gardner L. An interactive web-based dashboard to track COVID-19 in real time. The Lancet Infectious Diseases 2020;20(5):533–534.
26. Zhou T, Ji Y. Semiparametric Bayesian Inference for the Transmission Dynamics of COVID-19 with a State-Space Model. arXiv preprint arXiv:200605581 2020;.
27. Toda AA. Susceptible-infected-recovered (SIR) Dynamics of COVID-19 and Economic Impact. arXiv preprint arXiv:200311221 2020;.
28. Tashman L. Out-of sample tests of forecasting accuracy: A tutorial and review. International Journal of Forecasting 2000 01;16.
29. Chowell G, Simonsen L, Viboud C, Kuang Y. Is West Africa approaching a catastrophic phase or is the 2014 Ebola epidemic slowing down? Different models yield different answers for Liberia. PLoS Currents 2014;6.
30. Pell B, Kuang Y, Viboud C, Chowell G. Using phenomenological models for forecasting the 2015 Ebola challenge. Epidemics 2018;22:62–70.
31. Chowell G, Hincapie-Palacio D, Ospina J, Pell B, Tariq A, Dahal S, et al. Using phenomenological models to characterize transmissibility and forecast patterns and final burden of Zika epidemics. PLoS Currents 2016;8.
32. Chowell G, Luo R, Sun K, Roosa K, Tariq A, Viboud C. Real-time forecasting of epidemic trajectories using computational dynamic ensembles. Epidemics 2020;30:100379.
33. Chowell G. Fitting dynamic models to epidemic outbreaks with quantified uncertainty: A primer for parameter uncertainty, identifiability, and forecasts. Infectious Disease Modelling 2017;2(3):379–398.
34. Viboud C, Sun K, Gaffey R, Ajelli M, Fumanelli L, Merler S, et al. The RAPIDD ebola forecasting challenge: Synthesis and lessons learnt. Epidemics 2018;22:13–21.
35. Chowell G, Tariq A, Hyman JM. A novel sub-epidemic modeling framework for short-term forecasting epidemic waves. BMC Medicine 2019;17(1):164.
36. Jiang S, Zhou Q, Zhan X, Li Q. BayesSMILES: Bayesian Segmentation Modeling for Longitudinal Epidemiological

Studies. medRxiv 2020;https://www.medrxiv.org/content/early/2020/10/08/2020.10.06.20208132.1.
37. Gelman A, et al. Prior distributions for variance parameters in hierarchical models (comment on article by Browne and Draper). Bayesian Analysis 2006;1(3):515–534.
38. Haberman R. Mathematical models: Mechanical vibrations, population dynamics, and traffic flow. Society for Industrial and Applied Mathematics; 1998.
39. Werker A, Jaggard K. Modelling Asymmetrical Growth Curves that Rise and then Fall: Applications to Foliage Dynamics of Sugar Beet (Beta vulgarisL.). Annals of Botany 1997;79(6):657–665.
40. Desta F, Mac Siurtain M, Colbert J. Parameter estimation of nonlinear growth models in forestry. Silv Fenn 1999;33(4):327–336.
41. Kaps M, Herring W, Lamberson W. Genetic and environmental parameters for traits derived from the Brody growth curve and their relationships with weaning weight in Angus cattle. Journal of Animal Science 2000;78(6):1436–1442.
42. Topal M, Bolukbasi Ş. Comparison of nonlinear growth curve models in broiler chickens. Journal of Applied Animal Research 2008;34(2):149–152.
43. Hsieh YH, Lee JY, Chang HL. SARS epidemiology modeling. Emerging Infectious Diseases 2004;10(6):1165.
44. Hsieh YH. Richards model: A simple procedure for real-time prediction of outbreak severity. In: Modeling and Dynamics of Infectious Diseases World Scientific; 2009.p. 216–236.
45. Hsieh YH, Ma S. Intervention measures, turning point, and reproduction number for dengue, Singapore, 2005. The American Journal of Tropical Medicine and Hygiene 2009;80(1):66–71.
46. Hsieh YH, Chen C. Turning points, reproduction number, and impact of climatological events for multi-wave dengue outbreaks. Tropical Medicine & International Health 2009;14(6):628–638.
47. Hsieh YH. Pandemic influenza A (H1N1) during winter influenza season in the southern hemisphere. Influenza and Other Respiratory Viruses 2010;4(4):187–197.
48. Lee SY, Lei B, Mallick B. Estimation of COVID-19 spread curves integrating global data and borrowing information. PLOS ONE 2020 07;15(7):1–17. https://doi.org/10.1371/journal.pone.0236860.
49. Viboud C, Simonsen L, Chowell G. A generalized-growth model to characterize the early ascending phase of infectious disease outbreaks. Epidemics 2016;15:27–37.
50. Kermack WO, McKendrick AG. A contribution to the mathematical theory of epidemics. Proceedings of the Royal Society of London Series A 1927;115(772):700–721.
51. Bailey NT, et al. The mathematical theory of infectious diseases and its applications. Charles Griffin & Company Ltd, 5a Crendon Street, High Wycombe, Bucks HP13 6LE.; 1975.
52. Hethcote HW. The mathematics of infectious diseases. SIAM Review 2000;42(4):599–653.
53. Siettos CI, Russo L. Mathematical modeling of infectious disease dynamics. Virulence 2013;4(4):295–306.
54. Anastassopoulou C, Russo L, Tsakris A, Siettos C. Data-based analysis, modelling and forecasting of the COVID-19 outbreak. PloS One 2020;15(3):e0230405.
55. Pedersen MG, Meneghini M. Quantifying undetected COVID-19 cases and effects of containment measures in Italy. ResearchGate Preprint (online 21 March 2020) DOI 2020;10.
56. Weitz JS, Beckett SJ, Coenen AR, Demory D, Dominguez-Mirazo M, Dushoff J, et al. Modeling shield immunity to reduce COVID-19 epidemic spread. Nature Medicine 2020;p. 1–6.

Manuscript Click here to access/download;Manuscript;main.pdf

OXFORD (GIGA)^n SCIENCE

GigaScience, 2017, 1–??

doi: xx.xxxx/xxxx
Manuscript in Preparation
Paper

PAPER

Evaluating Short-term Forecasting among Different Epidemiological Models under a Bayesian Framework

Qiwei Li[1,†,*], Tejasv Bedi[1,†], Christoph U. Lehmann[2], Guanghua Xiao[2] and Yang Xie[2]

[1]The University of Texas at Dallas, Richardson, TX 75080, USA and [2]The University of Texas Southwestern Medical Center, Dallas, TX 75390, USA

[*]qiwei.li@utdallas.edu
[†]Contributed equally.

Abstract

Background: Forecasting of COVID-19 daily confirmed cases has been one of the several challenges posed to the governments and health sectors on a global scale. To facilitate informed public health decisions, the concerned parties rely on short-term daily projections generated via predictive modeling. We calibrate stochastic variants of growth models and the standard SIR model into one Bayesian framework to evaluate their short-term forecasts. **Results:** We implement rolling-origin cross-validation to compare the short-term forecasting performance of the stochastic epidemiological models and the autoregressive moving average model across the top 20 countries with the most confirmed cases as of August 22, 2020. **Conclusion:** In summary, it was noted that none of the models proved to be golden standards across all the regions in their entirety, while all outperformed the autoregressive moving average model in terms of the accuracy of forecast and interpretability.

Key words: COVID-19; SARS-CoV-2; Stochastic growth model; Stochastic SIR model; Time-series cross-validation.

Background

COVID-19, a respiratory disease coronavirus SARS-CoV-2, has rapidly created an ongoing global pandemic. It has become one of the leading causes of deaths in the United States (U.S.) and continues to spread fast in most other countries. Given the extent of the physical and economic suffering caused by the pandemic, there is an urgent public health need to better predict the spread of COVID-19 locally, nationally and globally. Since the emergence of the COVID-19 outbreak, a myriad of predictive modeling approaches have been proposed to predict trends of the disease to allow public health officials to develop effective policies and measures to suppress spread and minimize casualties. The five general approaches to forecast the number of new cases or the expected total mortality caused by the COVID-19 exist: 1) time-series forecasting such as autoregressive integrated moving average (ARIMA) [? ?]; 2) growth curve fitting based on the generalized Richards curve (GRC) or its special cases [? ? ? ? ?]; 3) compartmental modeling based on the susceptible-infectious-removed (SIR) models or their derivations [? ? ? ? ? ? ? ? ? ? ?]; 4) agent-based modeling [?]; 5) artificial intelligence (AI)-inspired modeling [? ? ? ?].

Each approach, whether deterministic or stochastic, has its own strengths. For instance, the ARIMA model combines the regressive process and the moving average, allowing to predict a given time series by considering its own lags and lagged forecast error. Curve fitting approaches (also known as phenomenological modeling) fit a curve to the observed number of cumulative confirmed cases or deaths with a certain error structure (e.g. Gaussian or Poisson), enabling meaningful interpre-

Compiled on: November 9, 2020.
Draft manuscript prepared by the author.

tation through curve parameters while accounting for measurement errors. Compartmental modeling (also known as mechanistic modeling) approaches assign partitions of the population to compartments corresponding to different stages of the disease and characterize the disease transmission dynamics by the flow of individuals through compartments. Agent-based modeling approaches use computer simulations to study the dynamic interactions among the agents (e.g. people in epidemiology) and between an agent and the environment. AI-based modeling approaches usually combines time series, clustering, and forecasting, resulting in an exemplary predictive performance. There has been a growing debate among researchers over model performance evaluation and how to find the best model appropriate for a certain feature (cases, deaths, etc.), a particular regional level (county, state, country, etc.), and other parameters. Fair evaluation and comparison of the output of different forecasting methods have remained elusive [?] since models vary in their complexity and the number of variables and parameters that characterize the dynamic states of a system.

Although predictive models for infectious diseases have been compared in the literature, to our best knowledge, existing work does not systematically compare performances, specifically with the same amount of available information. We calibrate stochastic variants of six different growth models (i.e. logistic, generalized logistic, Richards, generalized Richards, Bertalanffy, and Gompertz) and the standard SIR model, all of which can be specified by an ordinary differential equation(ODE), into one flexible Bayesian modeling framework. The main reason for limiting our analysis to these two modeling approaches is that both not only produce good short and long-term forecasts, but also provide useful insights to understand the disease dynamics of COVID-19. The growth models provide an empirical approach without a specific theory on the mechanisms that give rise to the observed patterns in the cumulative infection data, while the compartmental models incorporate key mechanisms involved in the disease transmission dynamics to explain patterns in the observed data.

In our Bayesian modeling framework, the bottom-level is represented by a negative binomial model that directly models infection count data and accounts for the over-dispersed observational errors. The top-level is derived from a choice of growth or compartmental models that characterizes a certain disease transmission dynamic through ODE(s). The Markov chain Monte Carlo (MCMC) algorithm is used to sample from the posterior distribution. The short-term forecasts are made from the resulting MCMC samples. We perform the rolling-origin cross-validation procedure to compare the prediction error of different stochastic models. In terms of regions, we used the top 20 countries in terms of confirmed case numbers for a country-level analysis. Observations included that 1) as the models learned more, the predictive performance improved in general for all regions; 2) none of the models proved to be the golden standard across all the regions, while the ARIMA model underperformed all stochastic models proposed in the paper. We designed a graphical interface that allows users to interact with future trends of COVID-19 at different geographic locations in the U.S. based on the real-time COVID-19 data. This web portal is updated daily and used to inform local policy-makers and the general public (https://qiwei.shinyapps.io/PredictCOVID19/).

Data Description

Let $\mathbf{C} = (C_1, \ldots, C_T)$ be a sequence of cumulative confirmed case numbers observed at T successive equally spaced points in time (e.g. day) in a specific region, where each entry $C_t \in \mathbb{N}$ for $t = 1, \ldots, T$. Further let C_0 be the initial value and $\dot{\mathbf{C}} = (\dot{C}_1, \ldots, \dot{C}_T)$ be the lag one difference of $\mathbf{C}$, where $\dot{C}_1 = C_1 - C_0$ and each following entry $\dot{C}_t = C_t - C_{t-1}, t = 2, \ldots, T$, i.e. the difference between two adjacent observations. In the analysis and modeling of a series of infectious disease daily report data, the time-series data could also be the cumulative death numbers, recovery case numbers, or their sums, denoted by $\mathbf{D}$ (Death), $\mathbf{E}$ (Recovery), and $\mathbf{R}$ (Removed), and their corresponding new case numbers, denoted by $\dot{\mathbf{D}}$, $\dot{\mathbf{E}}$, and $\dot{\mathbf{R}}$. Assuming a closed population with size N, the time-series data could also be the number of susceptible people, denoted by $\mathbf{S}$, with each entry $S_t = N - C_t$. In reality, only confirmed cases and deaths are reported in most regions. Recovery data are not available or suffer from under-reporting issues even if existing. Thus, our main goal is to make predictions of the future trend of an infectious diseases only based on the daily confirmed cases $\dot{\mathbf{C}}$.

Analyses

In this section, we discuss the findings of COVID-19 data analysis. We first implemented each of the growth models listed in Table ?? and the standard SIR model under the proposed Bayesian framework for the top 20 countries with the most confirmed cases as of August 22, 2020. Note that the input data was the sequence of daily confirmed cases $\dot{\mathbf{C}}$ only, which is accessible from the Johns Hopkins University Center for Systems Science and Engineering COVID-19 Data Repository (https://github.com/CSSEGISandData/COVID-19/). Several recent COVID-19 studies also based their analyses on this resource [? ? ?]. For our MCMC algorithms, we set 100, 000 iterations with the first half as burn in and chose weakly informative priors. Both numerical and graphical summaries for posterior inference and short-term forecasting are presented. Our final goal is to compare the predictive performance of all models, taking ARIMA as a benchmark model.

Forecasting of daily confirmed cases in the U.S.

We first present the forecasting of the U.S. daily confirmed cases made by the ARIMA and our Bayesian framework with the choices of a GRC or SIR model. As we can see from Figures ??, the GRC model demonstrates a downwards trend, the SIR model displays an upward trend, while the ARIMA model predicts a flat trajectory of daily predicted cases. A natural feature of epidemiological interest is the estimated final size and date of the epidemic. Growth models comprise of a model parameter K that estimates the final epidemic size. On the other hand, for the SIR model, there is no available parameter that estimates the final size. Hence, the final case count is approximated to be the predictive mean that converges to a certain value from the related MCMC samples. A similar strategy is applied to obtain the predicted mean of the final case counts using the ARIMA model [?]. The estimated cumulative confirmed cases by the end of 2020 are projected to be 13.1, 106.1, and 10.0 (in millions), fitting the GRC, SIR, and ARIMA models, respectively. Under the assumption that the epidemic lasts until the end of 2021, the final epidemic sizes are predicted to be 13.4, 187.3, and 22.0 (in millions) by the three models, respectively. To account for the reasons behind the discrepancy in forecasts and to measure the validity of the results, there is a need for an appropriate strategy to evaluate and compare the predictive performance of the concerned models.

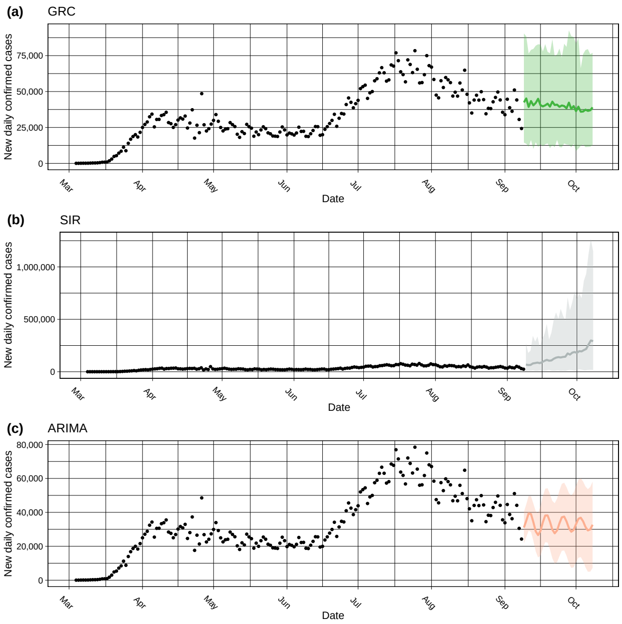

Figure 1. The one-month forecasting of new daily confirmed cases in the U.S. made by the (a) GRC and (b) SIR model under the proposed Bayesian framework, as well as the benchmark (c) ARIMA model. The black circles represent the observed case numbers since the beginning of March, 2020, while the colored circles and ribbons represent the predicted means and 95% prediction intervals, respectively.

Model comparison through rolling-origin cross-validation

Cross-validation (CV) is a resampling procedure used to evaluate regression and classification models on a limited data sample. The procedure randomly splits all data samples into two parts: training and testing sets, where the former is used to fit a model and the latter is used to evaluate the model's prediction performance in terms of a certain error measure. The key assumption of CV is that all data points should be independent and identically distributed (i.i.d.). Unfortunately, time-series data is serially autocorrelated i.e. the observations are dependent on the time they were recorded on. To circumvent this situation, the rolling-origin CV (ROCV) technique was proposed [?]. It splits the data into training and testing sets without hampering the i.i.d. assumption. An adaptation of this method is used here to evaluate the short-term forecasting performance among different top-level choices under the proposed Bayesian framework and ARIMA. Figure ?? shows the ROCV representation for an example of time-series data ($T = 17$). In our analysis, the choice of initial training sample size was set to seven days so as to evaluate how well the models are able to generate forecasts during the initial phase of the pandemic, while the testing sample size was chosen to be three days to meet with our objective of comparing short-term forecasting performance. We define the first day $t = 1$ as the date when the 100-th case was confirmed, so it varied for different countries.

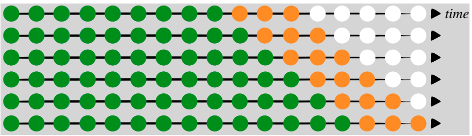

Figure 2. A visual guide to rolling-origin cross-validation (ROCV), where the total sample size $T = 17$, the initial training sample size is 9, and the testing sample size is 3. The green, orange, and white circles are training, testing, and unused samples in one CV iteration.

Algorithm 1 Rolling-origin cross-validation (ROCV)

Store the data starting day 1 to day T
Initialize the number of initial training observations k ($k = 7$)
Set the size of the testing set ω ($\omega = 3$)
while $k + \omega \leq T$ **do**
  Learn the first k observations (green circles) as training data
  Hold out the next $k+1, \ldots, k+\omega$ observations (orange circles) as the testing data
  Discard the remaining $T - (k+\omega)$ observations (white circles)
  Compute an out-of-bag prediction error measure on the testing set (orange circles)

$$\text{sMAPE} = \frac{2}{\omega} \sum_{t=k+1}^{k+\omega} \left| \frac{e_t}{\hat{y}_t + y_t} \right|$$

  $k = k + 1$
end while

A CV algorithm needs a predictive error metric that could quantify model performance in terms of forecasting accuracy. *Root mean square error* (RMSE) and *mean absolute deviations* (MAD) are candidates of error measures for out-of-bag predictions but are dependent on scale. As a result, large values may influence the errors to be larger. *Mean absolute percentage error* (MAPE) has been a widely used predictive measure due to its interpretability and its independence from scale. Although, the distribution of such percentage errors can be skewed if the data consists of values close to zero. Moreover, there is a possibility of this measure being undefined by having a zero in the denominator. To address these issues, an improved percentage error metric namely, *symmetric mean absolute percentage error* (sMAPE) was proposed [?]. This metric was considered in our analysis as it circumvented the problem of having an undefined measure and provided better symmetry as compared to MAPE. In all, we summarize the evaluation procedure used in this paper as follows.

Figure ?? displays the smoothed sMAPE curves generated by the ROCV across time for the top 20 countries with respect to the highest cumulative confirmed cases as of August 22, 2020. As we can see, all the models performed poorly in the early stage but as more and more data became available to be learned, the predictive performance gradually improved as the sMAPE dropped. It can also be observed that the ARIMA and SIR models were performing a lot worse in general than the growth models in the early phase. This could be attributed to the fact that ARIMA not having the growth specific parameters, unable to detect the early growth. On the other hand, making assumptions of a fixed transmission rate γ and due to the under-reported data issue, SIR performed poorly. While, the stochastic growth curves were able to learn the trend of epidemiological data in the initial phase with the help of the growth and scaling parameters. Although, towards the latter half of the epidemic, all the models were performing equally well. Hence, it is hard to conclude that any one particular dominated the entire duration of the epidemic.

Now, the question arises of whether we could pick one model which has the best predictive performance on an average for any particular country. To answer that we construct a Cleveland dot plot, as shown in Figure ??, that allows us to rank the model performance averaged over the entire duration, per country. Furthermore, the countries are arranged in de-

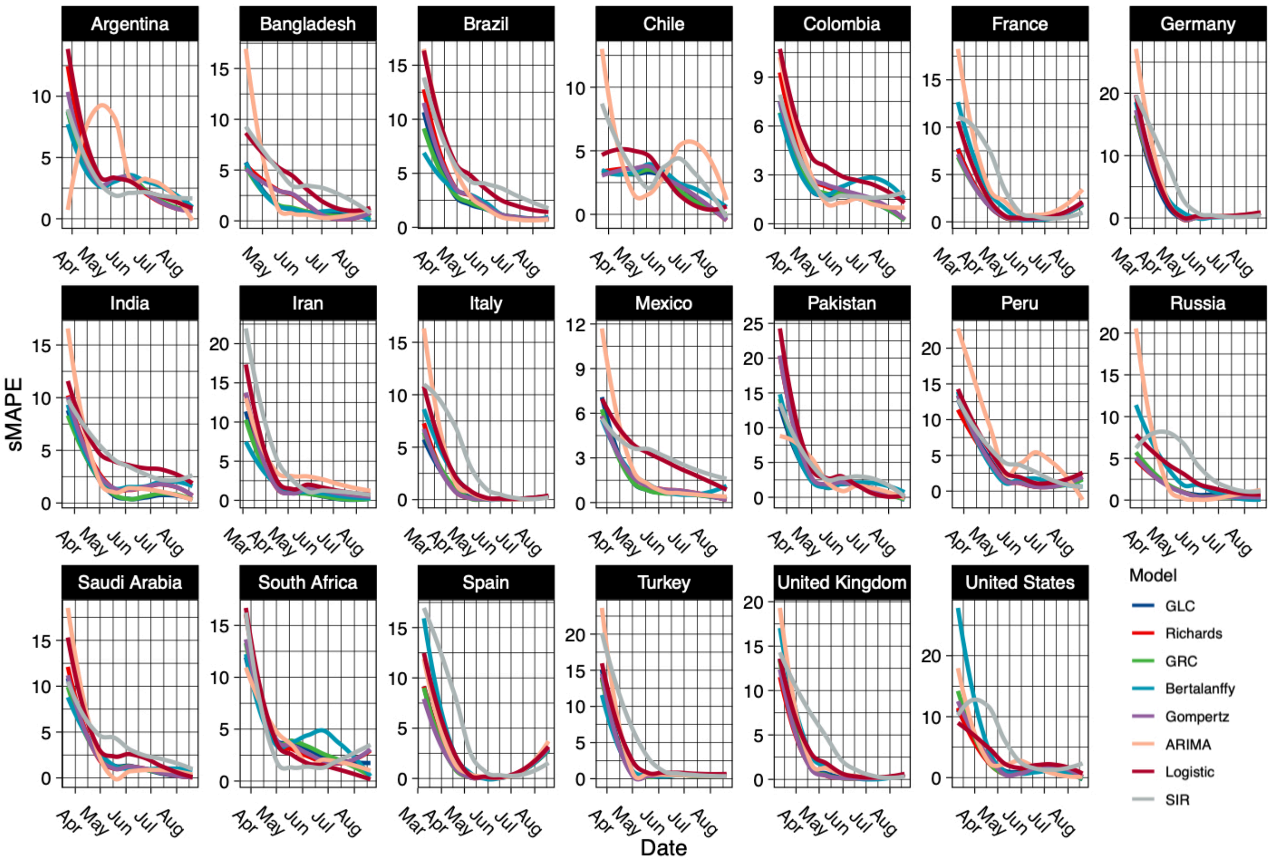

Figure 3. The smoothed sMAPE curves generated by the rolling-origin cross-validation (ROCV) over time for the top 20 countries with the most confirmed cases as of August 22, 2020.

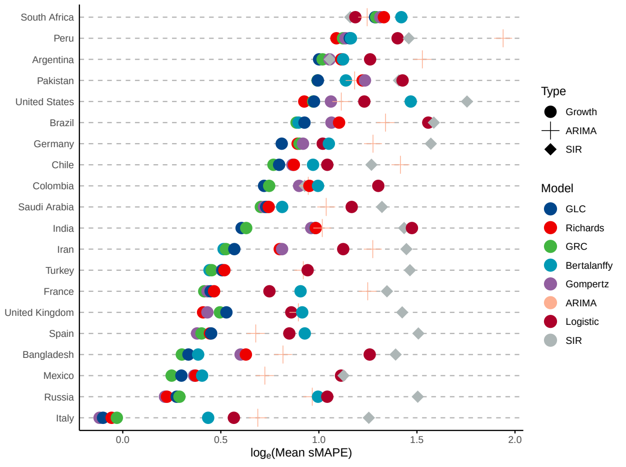

Figure 4. The Cleveland dot plot of the averaged sMAPE generated by the rolling-origin cross-validation (ROCV) for the top 20 countries with the most confirmed cases as of August 22, 2020.

scending order of predictive performance from the bottom to the top.

Discussion

It is observed that all the models had the best and the worst predictive performances for Italy and South Africa respectively. The Richards model had the minimum averaged sMAPE for forecasting cumulative case counts in the U.S. While, the GRC model had the lowest averaged sMAPE across seven countries followed by the GLC model with four. The SIR model was the best performer for South Africa. The Richards, Bertalanffy, and Gompertz models also had their fair share of predictive dominance in the remaining countries. On the other hand, the ARIMA model was a below-average performer across all countries.

In general, the GRC and GLC models were consistent performers throughout all countries due to their ability to detect sub-exponential growth rates at an early stage of an epidemic. In most cases, the GRC and Richards models were the best performers in countries that did not have symmetric 'S'-shaped growth patterns and displayed randomness as well as multiple peaks. This is due to the inclusion of the scale parameter α that could account for any asymmetry in the data. Countries including the U.S., Peru, Saudi Arabia, Iran, Turkey, and France display multiple peaks in the daily confirmed case counts. As a result, the Richards model performs the best in the U.S., United Kingdom (U.K.), and Peru, while the GRC model dominates in the rest of the countries having multiple peaks. Moreover, a random structure was observed in countries like Brazil, Chile, Bangladesh, and Mexico. GRC being the most complex model out of all the other growth models performed the best in these countries. On the other hand, the GLC model usually performed better in countries that had a single peak and attributed an approximate 'S'-shaped curvature. The GLC model was able to generalize better than the GRC model when the data was well structured and had less randomness. Countries including Argentina, Pakistan, Germany, Colombia, and India attributed a single peak without much randomness. As a result, the GLC model was a better performer in these countries. Whereas, in the case of South Africa, the usual growth models performed

the worst due to a staggering growth rate in the initial and the middle phase of the epidemic. The SIR model performed the best out of the worst while the logistic model performed well due to its simplicity. On the other hand, the Gompertz model was the best performer in Russia, Spain, and Italy as it generalizes better than the other models.

Conclusion

In this paper, we developed a number of stochastic variants of growth and compartmental models under a unified Bayesian framework. A theoretical comparison of growth models has been discussed in greater detail in the literature [? ? ? ? ? ? ? ?]. However, to our best knowledge, no work systematically compares their performances between all pairs as well as against a compartmental model such as the SIR model and a time-series forecasting model such as the ARIMA. Based on our analysis, we conclude that the proposed Bayesian framework not only allows room for interpretation but also offers an exemplary predictive performance when it comes to COVID-19 daily report data. Moreover, ARIMA being a pure learning algorithm is not able to match with the forecasting accuracy of stochastic models, let alone the model parameters of ARIMA do not provide any information on epidemiological interests.

For future work, we aim to develop an ensemble model, which can aggregate the prediction of each base model and results in once final prediction for the unseen data. Note that a group of researchers have recently introduced a GGM-GLM ensemble model [?] and compared forecasting performances of that with the individual models for the Ebola Forecasting Challenge [?]. It was reported that the ensemble model outperformed the others under some circumstances. We also plan to perform long-term forecasting evaluation using some epidemic features described in [?]. A sub-epidemic wave model that could detect multiple peaks in the data has been recently developed [?], which has the potential to improve forecasting performance. Thus, developing stochastic growth models via the addition of a change-point detection mechanism to account for multiple peaks is worth investigating. In this regard, we have demonstrated that an approach that combines a change-point detection model and a stochastic SIR model could significantly improve the short-term forecasting of the new daily confirmed cases [?].

Potential Implications

The proposed Bayesian epidemiological models in a unified framework lay the foundation of an integrative approach to model and predict epidemiological data with tremendous accuracy and interpretability. Growth and compartmental models obtained as solutions to ordinary differential equations (ODEs) are implemented to model epidemiological data under a deterministic setting as they provide a natural framework representative of such data types. However, the estimated model parameters crucial for providing insights into the nature of the epidemic are unreliable under the deterministic setting due to identifiability issues. The stochastic models mimic the structure of epidemiological models and incorporate parameter specific priors and measurement error to solve these issues. Researchers can follow a similar setup to predict cases and deaths caused by an epidemic at any geographical level given the availability of data. Furthermore, the stochastic SIR model can be augmented by incorporating mobility, hospitalization, and recovery data resulting in better forecasts. This work also promotes an algorithmic strategy to measure forecasting performances of time series models in general.

On a much broader scale, this work encourages researchers to exploring probabilistic approaches to model epidemiological data as well as developing computationally efficient algorithms that further meet time and cost constraints.

Methods

In this section, we present a bi-level Bayesian framework for predicting new daily confirmed cases during a pandemic in a closed society. The bottom level directly models the observed counts while accounting for measurement errors. Two alternatives for the top level are then introduced, both of which characterizes the epidemic dynamics through growth curve or compartmental trajectories, respectively. Before introducing the main components, we summarize the possibly observable data as follows.

Bottom-level: Time-series count generating process

We consider the new case number observed at time t, i.e. $\dot{C}_t$, are sampled from a negative binomial (NB) model,

$$\dot{C}_t \sim \mathrm{NB}(g(C_{t-1}, \Theta), \phi), \quad t = 2, \dots, T$$

as it automatically accounts for measurement errors and uncertainties associated with the counts. Here, we use $\mathrm{NB}(\mu, \phi), \mu, \phi > 0$ to denote a NB distribution with expectation μ and dispersion $1/\phi$. We assume this stochastic process is a Markov process, where the present state (at time t) depends only upon its previous state (at time $t-1$). Therefore, the NB mean is a function, denoted by $g(\cdot)$, of the case number observed at time $t-1$, characterized by a set of interpretable/uninterpretable model parameters Θ. With this parameterization, the NB variance is $\mu + \mu^2/\phi$, indicating that ϕ controls the variance of measurement error. A small value leads to a large variance to mean ratio, while a large value approaching infinity reduces the NB model to a Poisson model with the same mean and variance. The probability mass function of a NB random variable Y is $\frac{\Gamma(Y+\phi)}{Y!\Gamma(\phi)}\left(\frac{\phi}{\lambda+\phi}\right)^{\phi}\left(\frac{\lambda}{\lambda+\phi}\right)^{Y}$. Thus, we can write the full data likelihood as

$$f(\dot{\mathbf{C}}|\Theta, \phi) = \prod_{t=2}^{T} \frac{\Gamma(\dot{C}_t + \phi)}{\dot{C}_t!\Gamma(\phi)} \left(\frac{\phi}{g(C_{t-1}, \Theta) + \phi}\right)^{\phi} \left(\frac{g(C_{t-1}, \Theta)}{g(C_{t-1}, \Theta) + \phi}\right)^{\dot{C}_t}. \quad (1)$$

For the prior distribution of the dispersion parameter ϕ, we choose a gamma distribution, $\phi \sim \mathrm{Ga}(a_\phi, b_\phi)$. We recommend small values, such as $a_\phi = b_\phi = 0.001$, for a non-informative setting [?]. Note that the proposed framework can be also viewed as a stochastic discrete-time state-space model with a negative binomial error structure. The proposed Bayesian models, on average, mimics the epidemic dynamics and is more flexible than those deterministic epidemiological models, as it accounts for measurement error and has the potential to incorporate existing information into the prior structure of Θ.

Top-level I: Growth model

We first discuss the choices of $g(\cdot)$ when implementing growth models. The development of a variety of growth curves originates from population dynamics [?] and growth of biological systems [? ? ? ?] modeling. A number of growth curves have been adapted in epidemiology for trend characterization and forecasting of an epidemic, such as the severe acute respi-

ratory syndrome (SARS) [? ?], dengue fever [? ?], pandemic influenza A (H1N1) [?], Ebola virus disease (EVD) [? ?], Zika fever [?], and COVID-19 [? ? ? ?].

The underlying assumption is that the rate of growth of a population, organism, or infectious individuals eventually declines with size. The logistic curve (also known as sigmoid curve) is the simplest growth curve of continuous time $u \in \mathbb{R}$. It is a non-negative symmetric 'S'-shaped curve with equation $y(u) = \frac{K}{1+\exp(-\lambda(u-u_0))}$, where u_0 is the midpoint, K is the maximum value, and λ reflects the steepness of the curve. It is clear to see that $y(u)$ approaches K when $u \to \infty$, while it converges to zero when $u \to -\infty$. In fact, the continuous curve $y(u)$ is the solution of a first-order non-linear ODE,

$$\frac{dy(u)}{du} = \lambda y(u)\left(1 - \frac{y(u)}{K}\right)$$

with condition $y(u_0) = K/2$, where $dy(u)/du$ can be interpreted as time-variant growth rate of the curve y. The above ODE reveals: 1) a non-negative growth rate, $dy(u)/du > 0$ as $y(u) \in [0, K]$; 2) an approximately exponential growth at the initial stage, $y(u) \approx \exp(\lambda u)$ as $dy(u)/du \approx \lambda y(u)$ when $y(u) \to 0$; 3) no growth at the final stage, $y(u)\ dy(u)/du = 0$ when $y(u) \to K$; 4) a maximum growth rate of $\lambda K/4$ occurred when $y(u) = K/2$, indicated by $d^2y(u)/du^2 = \lambda dy(u)/du\,(1 - 2y(u)/K)$. Based on those curve characteristics, we can use the growth curve to characterize the trend of cumulative confirmed cases $\mathbf{C}$.

In this paper, we mainly consider a family of growth curves that are derived from the generalized Richards curve (GRC), which is the solution to the following ODE,

$$\frac{dy(u)}{du} = \lambda y(u)^p \left[1 - \left(\frac{y(u)}{K}\right)^\alpha\right]. \quad (2)$$

For those model-specific parameters in the context of epidemiology, K is the final epidemic size and should be an integer in the range of $(0, N]$, where N is the total population, $\lambda \in \mathbb{R}^+$ is the infectious rate at early epidemic stage, $p \in (0,1)$ is known as scaling of growth, and $\alpha \in \mathbb{R}^+$ controls the curve symmetry. As our observed infectious disease data are usually counts collected at successive equally spaced discrete time points, we formulate the NB mean function $g(\cdot)$ based on the discrete version of (??),

$$g(C_{t-1}, \Theta = \{K, \lambda, p, \alpha\}) = \lambda C_{t-1}^p \left[1 - \left(\frac{C_{t-1}}{K}\right)^\alpha\right]. \quad (3)$$

Table ?? provides a list of $g(\cdot)$'s for growth curves with their characteristics. All the listed growth curves have been utilized and discussed in previous epidemiological studies. We include all of those choices in our framework excluding the last one, which is based on the generalized growth curve (GGC), because it lacks the final epidemic size K specification.

Without any existing information, we assume that K is from a discrete uniform distribution in its range and γ is from a gamma or a beta distribution, depending on the choice of growth curves. For instance, for both logistic and Gompertz curves, we assume $\gamma \sim \text{Beta}(a_\gamma, b_\gamma)$, a natural modeling choice for parameter value restricted to the $(0, 1)$ interval, and suggest to choose $a_\gamma = b_\gamma = 1$ for a uniform setting; otherwise, we place a gamma prior, i.e. $\gamma \sim \text{Ga}(a_\gamma = 0.001, b_\gamma = 0.001)$. For the choice of GRC and generalized logistic curve (GLC), the prior of p is chose to be $\text{Beta}(a_p = 1, b_p = 1)$. Lastly, we set $\alpha \sim \text{Ga}(a_\gamma = 0.001, b_\gamma = 0.001)$ for fitting a GRC or Richards curve.

Top-level II: Compartmental model

The susceptible-infected-removed (SIR) model was developed to simplify the mathematical modeling of human-to-human infectious diseases by ?]. It is a fundamental compartmental model in epidemiology. At any given time u, each individual in a closed population with size N is assigned to three distinctive compartments with labels: susceptible (S), infectious (I), or removed (R, being either recovery or dead). The standard SIR model describes the flow of people from S to I and then from I to R by the following set of nonlinear ODEs:

$$\begin{cases} \frac{dS(u)}{du} = -\beta S(u)\frac{I(u)}{N} \\ \frac{dI(u)}{du} = \beta S(u)\frac{I(u)}{N} - \gamma I(u) \\ \frac{dR(u)}{du} = \gamma I(u) \end{cases},$$

where $S(u)$, $I(u)$, and $R(u)$ are the population numbers of susceptible, infectious, and removed compartments measured in time u, subjecting to $S(u) + I(u) + R(u) = N, \forall u$. Another nature constraint is $dS(u)/du + dI(u)/du + dR(u)/du = 0$. Here, $\beta \in \mathbb{R}^+$ is the disease transmission rate, $\gamma \in \mathbb{R}^+$ is the removal rate, and their ratio $\mathcal{R}_0 = \beta/\gamma$ is defined as the *basic reproductive number*. The rationale behind the first equation is that the number of new infections during an infinitesimal amount of time, $-dS(u)/du$, is equal to the number of susceptible people, $S(u)$, times the product of the contact rate, $I(t)/N$, and the disease transmission rate β. The third equation reflects that the infectious individuals leave the infectious population per unit time, $dI(u)/du$, as a rate of $\gamma I(u)$. The second equation follows immediately from the first and third ones as a result of $dS(u)/du + dI(u)/du + dR(u)/du = 0$. Assuming that only a small fraction of the population is infected or removed in the onset phase of an epidemic, we have $S(u)/N \approx 1$ and thus the second equation reduces to $dI(u)/du = (\beta - \gamma)I(u)$, revealing that the infectious population is growing if and only if $\beta > \gamma$. As the expected lifetime of an infected case is given by γ^{-1}, the ratio $\mathcal{R}_0 = \beta/\gamma$ is the average number of new infectious cases directly produced by an infected case in a completely susceptible population. The so called basic reproductive number is a good indicator of the transmissibility of an infectious disease.

In this paper, we only consider the standard SIR model, although it is still feasible to design $g(\cdot)$'s from its variations (see a comprehensive summary [?]), such as the susceptible-infectious-susceptible (SIS) model, the susceptible-infectious-recovered-deceases (SIRD) model, the susceptible-exposed-infectious-removed (SEIR) model, the susceptible-exposed-infectious-susceptible (SEIS) model, and their versions with the maternally-derived immunity compartment [?], as well as the recently developed extended-SIR (eSIR) model [?]. For modeling discrete time-series data, we use the discrete-time version of the standard SIR model,

$$\begin{cases} \dot{S}_t = -\beta S_{t-1}\frac{I_{t-1}}{N} \\ \dot{I}_t = \beta S_{t-1}\frac{I_{t-1}}{N} - \gamma I_{t-1} \\ \dot{R}_t = \gamma I_{t-1} \end{cases}. \quad (4)$$

The model has three trajectories, one for each compartment. The compositional nature of the three trajectories implies that we only need two of the three sequence data, e.g. $S_t = N - C_t$ and R_t for $t = 1, \ldots, n$. However, recovery data only exist in few regions, and suffer from under-reporting issue even if existing, which makes both model inference and predictions infeasible. Alternatively, we consider both of the removed and actively infectious cases as missing data and mimic their relationship in spirit to some compartmental models in epidemiology. Specifically, we assume the number of new removed cases at time t, i.e. $\dot{R}_t$, is sampled from a Poisson distribution with mean γI_{t-1},

Table 1. List of $g(\cdot)$'s functions based on growth curves

Models	$g(C_{t-1}, \Theta)$	Parameters Θ	Curve $y(u)$	$y(u)$ at the turning point	Examples
GRC	$\lambda C_{t-1}^{p}\left[1-\left(\frac{C_{t-1}}{K}\right)^{\alpha}\right]$	$K \in \mathbb{N}$,<br>$\lambda \in \mathbb{R}^{+}$,<br>$p \in (0,1)$,<br>$\alpha \in \mathbb{R}^{+}$	N/A	$\left(\frac{p}{p+\alpha}\right)^{1/\alpha} K$	[? ? ?]
Richards	$\lambda C_{t-1}\left[1-\left(\frac{C_{t-1}}{K}\right)^{\alpha}\right]$	$K \in \mathbb{N}$,<br>$\lambda \in \mathbb{R}^{+}$,<br>$\alpha \in \mathbb{R}+$	$K\left(1+A\exp(-\lambda\alpha u)\right)^{-1/\alpha}$,<br>where $A=-1+\left(\frac{K}{y(0)}\right)^{\alpha}$	$\left(\frac{1}{1+\alpha}\right)^{1/\alpha} K$	[? ? ? ? ?]
GLC	$\lambda C_{t-1}^{p}\left(1-\frac{C_{t-1}}{K}\right)$	$K \in \mathbb{N}$,<br>$\lambda \in \mathbb{R}^{+}$,<br>$p \in (0,1)$	N/A	$\frac{p}{p+1}K$	[? ? ? ?]
Logistic	$\lambda C_{t-1}\left(1-\frac{C_{t-1}}{K}\right)$	$K \in \mathbb{N}$,<br>$\lambda \in (0,1)$	$K\left(1+A\exp(-\lambda u)\right)^{-1}$,<br>where $A=-1+\frac{K}{y(0)}$	$\frac{1}{2}K$	[? ? ? ? ?]
Bertalanffy	$\lambda C_{t-1}^{\frac{2}{3}}\left[1-\left(\frac{C_{t-1}}{K}\right)^{\frac{1}{3}}\right]$	$K \in \mathbb{N}$,<br>$\lambda \in \mathbb{R}^{+}$	$K\left(1+A\exp(-\frac{1}{3}\gamma K^{-1/3}u)\right)^{3}$,<br>where $A=1-\left(\frac{y(0)}{K}\right)^{1/3}$	$\frac{8}{27}K$	[?]
Gompertz	$\lambda C_{t-1}\log\frac{K}{C_{t-1}}$	$K \in \mathbb{N}$,<br>$\lambda \in (0,1)$	$K\exp(A\exp(-\lambda u))$,<br>where $A=\log\frac{y(0)}{K}$	$\frac{1}{e}K$	[?]
GGC	λC_{t-1}^{p}	$\lambda \in \mathbb{R}^{+}$,<br>$p \in (0,1)$	$(A+\lambda u(1-p))^{1/(1-p)}$<br>where $A=y(0)^{1-p}$	N/A	[? ? ? ? ?]

Abbreviations: GRC is generalized Richards curve; GLC is generalized logistic curve; GGC is generalized growth curve.

that is, $\dot{R}_t \sim \text{Poi}(\gamma I_{t-1}) = \text{Poi}(\gamma(N - C_{t-1} - R_{t-1}))$, where γ should be specified. Such a strategy but with different error structure was also considered in some other compartmental models in epidemiology [? ? ?]. We can estimate the value of γ from publicly available high-quality data where confirmed, deaths, and recovery cases are all well-documented, or from prior epidemic studies due to the same under-reporting issue in actual data. In this paper, we choose the removal rate $\gamma = 0.1$ as suggested by ?] and ?]. Based on this simplification, we rewrite the first equation in (??) as,

$$(N - C_t) - (N - C_{t-1}) \quad = \quad -\beta(N - C_{t-1})\frac{N - C_{t-1} - R_{t-1}}{N},$$

resulting in

$$\dot{C}_t \quad = \quad \beta(N - C_{t-1})\frac{N - C_{t-1} - R_{t-1}}{N}.$$

Thus, we formulate the NB mean function $g(\cdot)$ for the standard SIR model as,

$$g(C_{t-1}, \Theta = \{\beta\}|\boldsymbol{R}) \quad = \quad \beta(N - C_{t-1})\frac{N - C_{t-1} - R_{t-1}}{N}, \qquad (5)$$

where $\boldsymbol{R}$ can be sequentially inferred from $\boldsymbol{C}$.

Without any existing information, in our Bayesian framework we assume β from a gamma distribution with hyperparameters that makes both the mean and variance of the transformed variable $\mathcal{R}_0 = \beta/\gamma$ equal to 1, that is, $\beta \sim \text{Ga}(1, 1/\gamma)$.

Model Fitting

In this section, we briefly describe the MCMC algorithm for posterior inference and forecasting. Our Bayesian inferential strategy allows us to simultaneously infer all model-specific parameters and quantify their uncertainties.

MCMC algorithms

We first describe how to update the dispersion parameter ϕ in the proposed Bayesian framework, as it does not depend on the choice of models. At each MCMC iteration, we perform the following step:

Update of dispersion parameter ϕ**:** We update ϕ by using a random walk Metropolis-Hastings (RWMH) algorithm. We first propose a new ϕ^*, of which logarithmic value is generated from $\text{N}\left(\log\phi, \tau_\phi^2\right)$ and then accept the proposed value ϕ^* with probability $\min(1, m_{\text{MH}})$, where the Hastings ratio is

$$m_{\text{MH}} \quad = \quad \frac{f(\dot{\boldsymbol{C}}|\phi^*, \Theta)}{f(\dot{\boldsymbol{C}}|\phi, \Theta)}\frac{\pi(\phi^*)}{\pi(\phi)}\frac{J(\phi \leftarrow \phi^*)}{J(\phi^* \leftarrow \phi)}.$$

Here we use $J(\cdot \leftarrow \cdot)$ to denote the proposal probability distribution for the selected move. Note that the last term, which is the proposal density ratio, cancels out for this RWMH update.

Top-level as a growth model

We only present the updates of each parameters in the GRC model, as all other derivative models are its special cases. Our primary interest lies in the estimation of the final pandemic size K and the infectious rate at early epidemic stage λ.

Update of final epidemic size parameter K**:** We update K by using a RWMH algorithm. We first propose a new K^*, of which logarithmic value is generated from a truncated Poisson distribution $\text{Poi}(\log K)$ within $[\log C_T, \log N]$ and then accept the proposed value K^* with probability $\min(1, m_{\text{MH}})$, where the Hast-

ings ratio is

$$m_{\mathrm{MH}} = \frac{f(\dot{\mathbf{C}}|\phi, K^*, \lambda, p, \alpha)}{f(\dot{\mathbf{C}}|\phi, K, \lambda, p, \alpha)} \frac{\pi(K^*)}{\pi(K)} \frac{J(K \leftarrow K^*)}{J(K^* \leftarrow K)}.$$

Note that with a discrete uniform prior on K, the last two terms cancel out for this RWMH update.

Update of infectious rate parameter λ: We update λ by using a RWMH algorithm. We first propose a new λ^*, of which logarithmic value is generated from $\mathrm{N}\left(\log\lambda, \tau_\lambda^2\right)$ and then accept the proposed value λ^* with probability $\min(1, m_{\mathrm{MH}})$, where the Hastings ratio is

$$m_{\mathrm{MH}} = \frac{f(\dot{\mathbf{C}}|\phi, K, \lambda^*, p, \alpha)}{f(\dot{\mathbf{C}}|\phi, K, \lambda, p, \alpha)} \frac{\pi(\lambda^*)}{\pi(\lambda)} \frac{J(\lambda \leftarrow \lambda^*)}{J(\lambda^* \leftarrow \lambda)}.$$

Note that the last term, which is the proposal density ratio, cancels out for this RWMH update.

Update of growth scaling parameter p: We update p by using a RWMH algorithm. We first propose a new p^*, of which logarithmic value is generated from a truncated normal distribution $\mathrm{N}\left(\log p, \tau_p^2\right)$ within $[-\infty, 0]$ and then accept the proposed value p^* with probability $\min(1, m_{\mathrm{MH}})$, where the Hastings ratio is

$$m_{\mathrm{MH}} = \frac{f(\dot{\mathbf{C}}|\phi, K, \lambda, p^*, \alpha)}{f(\dot{\mathbf{C}}|\phi, K, \lambda, p, \alpha)} \frac{\pi(p^*)}{\pi(p)} \frac{J(p \leftarrow p^*)}{J(p^* \leftarrow p)}.$$

Note that with a uniform prior on p over its range $[0, 1]$, the last two terms cancel out for this RWMH update.

Update of symmetry parameter α: We update α by using a RWMH algorithm. We first propose a new α^*, of which logarithmic value is generated from $\mathrm{N}\left(\log\alpha, \tau_\alpha^2\right)$ and then accept the proposed value α^* with probability $\min(1, m_{\mathrm{MH}})$, where the Hastings ratio is

$$m_{\mathrm{MH}} = \frac{f(\dot{\mathbf{C}}|\phi, K, \lambda, p, \alpha^*)}{f(\dot{\mathbf{C}}|\phi, K, \lambda, p, \alpha)} \frac{\pi(\alpha^*)}{\pi(\alpha)} \frac{J(\alpha \leftarrow \alpha^*)}{J(\alpha^* \leftarrow \alpha)}.$$

Note that the last term, which is the proposal density ratio, cancels out for this RWMH update.

Top-level as a compartmental model

Our primary interest lies in the estimation of the reproductive number $\mathcal{R}_0 = \beta/\gamma$, which reflects the transmissibility of the disease. With our assumption that γ is given, we propose the following updates in each MCMC iterations.

Generate R based on C: We assume $I_1 = C_1$, i.e. all the confirmed cases are capable to pass the disease to all susceptible individuals in a closed population at the very beginning. In other words, $R_1 = 0$. Then we sample $R_2 \sim \mathrm{Poi}(\gamma I_1)$, where γ is a pre-specified tuning parameter. Due to the compositional nature, we can compute $I_2 = I_1 + \dot{C}_2 - R_2$, where $\dot{C}_2 = C_2 - C_1$ is the new confirmed cases and R_1 is the total removed cases from the actively infectious population at time 2. Next, we repeat this process of sampling $R_t \sim \mathrm{Poi}(\gamma I_{t-1})$ and computing $I_t = I_{t-1} + \dot{C}_t - R_t$, to generate the sequence $\mathbf{R}$.

Update of reproduction number parameter β: We update β by using a RWMH algorithm. We first propose a new β^*, of which logarithmic value is generated from a truncated normal distribution $\mathrm{N}\left(\log\beta, \tau_\beta^2\right)$ and then accept the proposed value β^* with probability $\min(1, m_{\mathrm{MH}})$, where the Hastings ratio is

$$m_{\mathrm{MH}} = \frac{f(\dot{\mathbf{C}}|\beta^*, \mathbf{R})}{f(\dot{\mathbf{C}}|\beta, \mathbf{R})} \frac{\pi(\beta^*)}{\pi(\beta)} \frac{J(\beta \leftarrow \beta^*)}{J(\beta^* \leftarrow \beta)}.$$

Note that the last term, which is the proposal density ratio, cancels out for this RWMH update.

Posterior inference

We obtain posterior inference by post-processing the MCMC samples after burn-in. Suppose that a sequence of MCMC samples on $\theta, \theta \in \{\phi, K, \lambda, p, \alpha, \beta\}$,

$$\theta^{(1)}, \ldots, \theta^{(U)}$$

have been collected, where $u, u = 1, \ldots, U$ indexes the iteration after burn-in. An approximate Bayesian estimator of each parameter can be simply obtained by averaging over the samples, $\hat{\theta} = \sum_{u=1}^{U} \theta^{(u)}/U$. In additional to that, a quantile estimation or credible interval for each parameter of interest can be obtained from this sequence as well.

Forecasting

Based on the sequences of MCMC samples on K, λ, p, and α in the growth model or β in the compartmental model, we can predict the cumulative or new confirmed cases at any future time T_f by Monte Carlo simulation. Particularly, from time $T+1$ to T_f, we sequentially generate

$$\dot{C}_t^{(u)} \sim \mathrm{NB}(g(C_{t-1}, \Theta^{(u)}), \phi^{(u)}), \quad t = T+1, \ldots, T_f. \qquad (6)$$

Then, both short and long-term forecast can be made by summarizing the $(T_f - T)$-by-U matrix of MCMC samples. For instance, the predictive number of cumulative and new confirmed cases at time $T+1$, in average, are $\sum_{u=1}^{U} C_{T+1}^{(u)}/U$ and $\sum_{u=1}^{U} \dot{C}_{T+1}^{(u)}/U$, respectively.

Software

This paper introduces a user-friendly interactive web application (`https://qiwei.shinyapps.io/PredictCOVID19/`) integrated with R `Shiny` package. `Shiny` is a web platform that allows users to interact with real-time data and use a myriad of visualization tools to analyze it. The web application has been developed to help the general public assess both short and long-term forecasts of COVID-19 across the U.S. at both state and metropolitan-level. The numbers of cumulative or new daily confirmed cases as well as deaths are projected by different growth models and the SIR model under the proposed Bayesian framework. Alongside the numerical summaries, users can view and interpret the trends that cover the same information. To validate the short-term forecasting, numerical and graphical summaries of MAE and MAPE of the predictions are provided for the more advanced users. Moving on to the long-term forecasting, the models estimate the peak number of cases and deaths as well as their respective dates. Moreover, a predictive estimate for the final size and date is also offered. Finally, for the users that are keen to visualize the currently observed cases at a geographical level, the website offers county-level spatial maps.

Availability of source code and requirements

- Project name: BayesEpiModels
- Project home page: `https://github.com/liqiwei2000/BayesEpiModels`
- Operating system(s): Windows and Linux

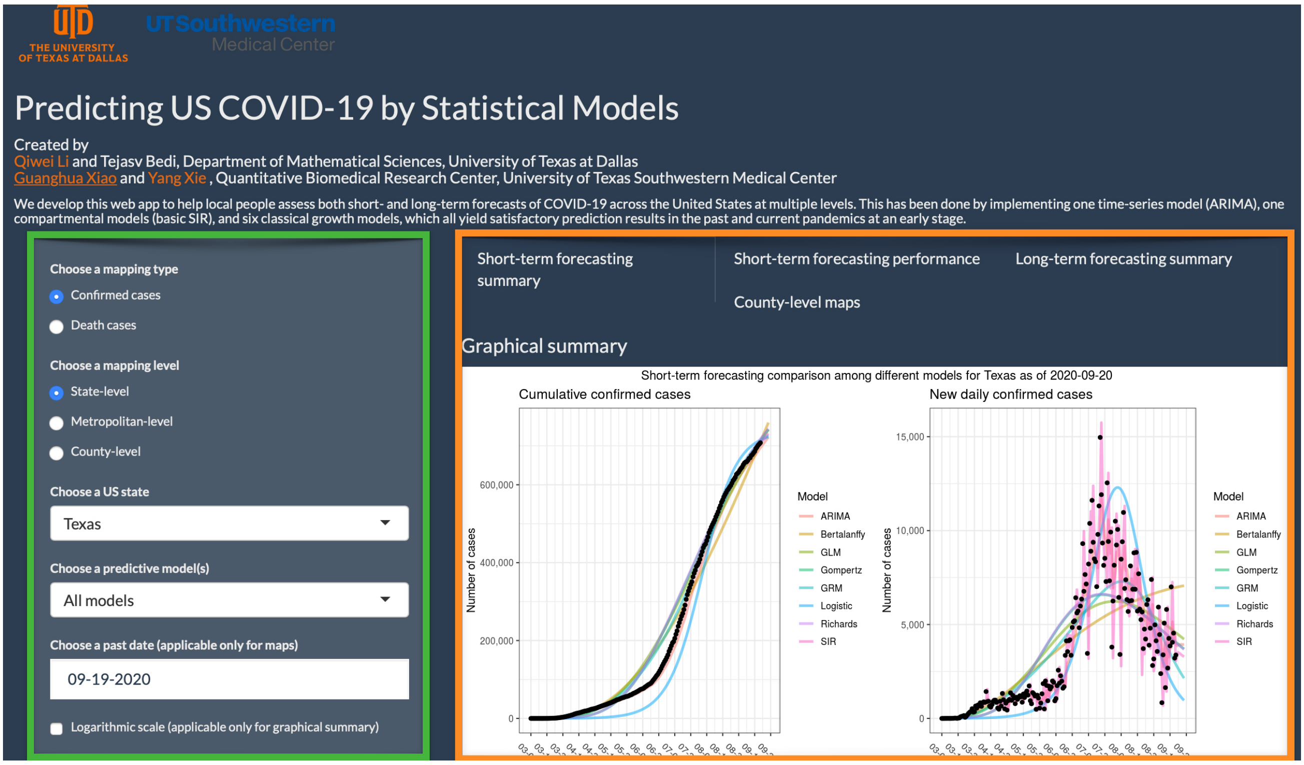

Figure 5. The web interface of the COVID-19 trend analysis page. The green box highlights the input panel that allows users to choose different mapping types and levels for a region. The orange box highlights the visualizations for short-term forecasting as per the instructions of users. Other tabs offer different graphs for summarizing the model performance, long-term forecasting, and county-level spatial maps.

- Programming language: R (version 3.6.0.)
- Other requirements: Not applicable.
- License: GNU General Public License v3.0

Availability of supporting data and materials

The related R/C++ codes for model preparation and execution are available on GitHub at https://github.com/liqiwei2000/BayesEpiModels. The R Shiny web application is available for users at https://qiwei.shinyapps.io/PredictCOVID19/. The COVID-19 data repository is operated by the Johns Hopkins University Center for Systems Science and Engineering (JHU CSSE) and is freely available on GitHub at https://github.com/CSSEGISandData/COVID-19/.

List of abbreviations

AI: Artificial Intelligence, ARIMA: Autoregressive integrated moving average, COVID-19: Coronavirus disease - 2019, CV: Cross-validation, eSIR: extended susceptible-infectious-removed, GGC: Generalized growth curve, GGM-GLM: Generalized growth model - Generalized logistic model, GLC: Generalized logistic curve, GRC: Generalized Richards curve, i.i.d.: independent and identically distributed, JHUCSSE: Johns Hopkins University Center for Systems Science and Engineering, MAD: Mean absolute deviations, MAPE: Mean absolute percentage error, MCMC: Markov chain Monte Carlo, NB: Negative Binomial, ODE: Ordinary differential equation, RMSE: Root mean square error, ROCV: Rolling-origin cross-validation, RWMH: Random walk Metropolis-Hastings, SARS-Cov-2: Severe acute respiratory syndrome coronavirus 2, SEIR: susceptible-exposed-infectious-removed, SEIS: susceptible-exposed-infectious-susceptible, SIR: susceptible-infectious-removed, SIRD: susceptible-infectious-recovered-deceases, SIS: susceptible-infectious-susceptible, sMAPE: symmetric Mean Absolute Percentage Error, U.K.: United Kingdom, U.S.: United States.

Ethical Approval

Not applicable.

Consent for publication

Not applicable.

Competing Interests

The authors declare that they have no competing interests.

Funding

This work was partially supported by the National Institutes of Health [R35GM136375, P30CA142543, 5R01CA152301, P50CA70907, 1R01GM115473]; and the Cancer Prevention and Research Institute of Texas [RP190107, RP180805].

Author's Contributions

QL has developed the Bayesian framework, designed the MCMC algorithms, and constructed the R Shiny web application. QL and TB have contributed to the review of different methods and collaborated in the real data analysis. QL, CL, GX, and YX have conceived the study and supervised the web application development and the statistical analyses. All authors have contributed to the writing of the manuscript. All authors have read and approved the final manuscript.

Acknowledgements

The authors would like to thank Drs. Swati Biswas, Min Chen, Pankaj Choudhary, and Vladimir Dragovic at the University of Texas at Dallas for their support and valuable suggestions on this work.
