# Supplementary material for: Evaluating short-term forecasting of COVID-19 cases among different epidemiological models under a Bayesian framework
Source: Gigascience. 2021 Feb 19;10(2):giab009. doi: 10.1093/gigascience/giab009 (PMC7928884; doi:10.1093/gigascience/giab009)
Supplement: giab009_Response_to_Reviewer_Comments_Original_Submission [file giab009_response_to_reviewer_comments_original_submission.pdf]

Author's Response To Reviewer Comments

Close

For a clean version of the response letter, please see the pdf file uploaded as the supplementary material.

We want to thank the editor and the two reviewers for this opportunity to revise our work. Based on the comments, we have performed a minor revision. The reviewers' comments have greatly helped us to improve the paper. We hope that the revised paper is now acceptable for publication in GigaScience. Below, we respond to each reviewer's points. All changes in the text of the paper have been highlighted in red.

Editor Comment 1: Please register any new software application in the bio.tools and SciCrunch.org databases to receive biotoolsID identifiers and Research Resource Identification Initiative ID (RRID) and include these in your manuscript. This will facilitate tracking, reproducibility and re-use of your tool.

Response: We have registered both the web application and source code associated with the paper in the bio.tools and SciCrunch.org databases and included these in the Software and Availability of source code and requirement sections on page 9.

Editor Comment 2: Please include other identifiers in the paper such as ORCID details of any authors that have them.

Response: The ORCID of Qiwei Li is 0000-0002-1020-3050, the ORCID of Tejasv Bedi is 0000- 0001-7532-4075, the ORCID of Christoph Lehmann is 0000-0001-9559-4646, the ORCID of Guanghua Xiao is 0000-0001-9387-9883, and the ORCID of Yang Xie is 0000-0001-9456-1762. We will update this information in the submission system.

Reviewer 1 Comment 1: Please, explain better the equation of sMAPE in Algorithm 1, defining all the variables (et, etc.).

Response: We are sorry for any confusion regarding the definition of sMAPE. We have rewritten the sMAPE in Algorithm 1 and enriched the Model comparison in the rolling-origin cross-validation section on page 3.

Reviewer 1 Comment 2: Please, discuss something regarding the possible discretization of Equation (2), (3), or (4). Specifically, Equation (4) seems still in continuous time since there are derivatives and not differences. Please, clarify this point.

Response: Thank you for the suggestion to clarify this point. As for Equation (2), we have given its discretized form right after the continuous case. As for Equation (3), the original version is already in discrete time. Thus, no change needs to be made. As for Equation (4), we have defined the notation ˙ as the forward difference operator to avoid any confusion with the derivative.

Reviewer 1 Comment 3: Regarding the MCMC and Metropolis part, in order to improve the state-of-the-art discussion, I suggest to discuss more sophisticated versions of the MH algorithm, such as the Multiple Try Metropolis algorithms and adaptive MCMC schemes (see e.g. Mira et al., 2001Haario et al., 2006Martino, 2018Liang et al., 2011).

Response: Thank you for your valuable suggestion. We have added a short discussion about several advanced versions of the Metropolis-Hastings algorithm that could potentially be used to improve our Markov chain Monte Carlo algorithms in the last paragraph of the Conclusion section on page 5.

Reviewer 2 Comment 1: Rightfully they note that "none of the models proved to be golden standards across all the regions" and I should add also across time scales where definitely a second and possibly a third wave have already been observed to develop. Although the authors briefly mention this in their

paper, I believe they should make it clearer why there is such a limitation for the models and their variants discussed herein.

Response: Thank you for your valuable suggestion. We are currently extending our work to account for multiple peaks. We have added a short discussion about the limitation of the single-peak-based modeling work in the last paragraph of the Conclusion section on page 5.

Close
