# Supplementary material for: Evaluating short-term forecasting of COVID-19 cases among different epidemiological models under a Bayesian framework
Source: Gigascience. 2021 Feb 19;10(2):giab009. doi: 10.1093/gigascience/giab009 (PMC7928884; doi:10.1093/gigascience/giab009)
Supplement: giab009_Response_to_Reviewer_Comments_Revision_1 [file giab009_response_to_reviewer_comments_revision_1.pdf]

Author's Response To Reviewer Comments

Close

We have carried out all final editings requested/suggested by the editor. Please see our point-to-point responses to the editor in the supplementary files "comments_main_v4.pdf." The updated .tex and .pdf files of the paper have been uploaded for the final check. We ask the editor's office to add the ORCID for all authors in the paper, as we do not know how to do that in the .tex file. The ORCID information can be found in our response in the supplementary files "comments_main_v4.pdf."

Close
